# International interlaboratory study comparing single organism 16S rRNA gene sequencing data: Beyond consensus sequence comparisons

**DOI:** 10.1016/j.bdq.2015.01.004

**Published:** 2015-03-05

**Authors:** Nathan D. Olson, Steven P. Lund, Justin M. Zook, Fabiola Rojas-Cornejo, Brian Beck, Carole Foy, Jim Huggett, Alexandra S. Whale, Zhiwei Sui, Anna Baoutina, Michael Dobeson, Lina Partis, Jayne B. Morrow

**Affiliations:** a Biosystems and Biomaterials Division, National Institute of Standards and Technology, 100 Bureau Dr, Gaithersburg, MD 20899, USA; b Statistical Engineering Division, National Institute of Standards and Technology, 100 Bureau Dr, Gaithersburg, MD 20899, USA; c Instituto de Salud Pública de Chile, Chile; d American Type Culture Collection, 10801 University Boulevard, Manassas, VA 20110, USA; e Science and Innovation Division, LGC, Queens Rd, Teddington, Middlesex TW11 0LY, UK; f National Institute of Metrology, Beijing 100013, China; g National Measurement Institute, West Lindfield, NSW 2070, Australia

**Keywords:** DNA sequencing, 16S rRNA, Interlaboratory study

## Abstract

This study presents the results from an interlaboratory sequencing study for which we developed a novel high-resolution method for comparing data from different sequencing platforms for a multi-copy, paralogous gene. The combination of PCR amplification and 16S ribosomal RNA gene (16S rRNA) sequencing has revolutionized bacteriology by enabling rapid identification, frequently without the need for culture. To assess variability between laboratories in sequencing 16S rRNA, six laboratories sequenced the gene encoding the 16S rRNA from *Escherichia coli* O157:H7 strain EDL933 and *Listeria monocytogenes* serovar 4b strain NCTC11994. Participants performed sequencing methods and protocols available in their laboratories: Sanger sequencing, Roche 454 pyrosequencing^®^, or Ion Torrent PGM^®^. The sequencing data were evaluated on three levels: (1) identity of biologically conserved position, (2) ratio of 16S rRNA gene copies featuring identified variants, and (3) the collection of variant combinations in a set of 16S rRNA gene copies. The same set of biologically conserved positions was identified for each sequencing method. Analytical methods using Bayesian and maximum likelihood statistics were developed to estimate variant copy ratios, which describe the ratio of nucleotides at each identified biologically variable position, as well as the likely set of variant combinations present in 16S rRNA gene copies. Our results indicate that estimated variant copy ratios at biologically variable positions were only reproducible for high throughput sequencing methods. Furthermore, the likely variant combination set was only reproducible with increased sequencing depth and longer read lengths. We also demonstrate novel methods for evaluating variable positions when comparing multi-copy gene sequence data from multiple laboratories generated using multiple sequencing technologies.

## Highlights

• Sequencing results were in agreement for biologically conserved positions.
• For biologically variable positions, sequencing depth impacted precision.
• Results were biased by the algorithm used to align reads to the reference.

## 1 Introduction

The 16S ribosomal RNA gene (16S rRNA) is the most commonly used marker in bacterial genotypic identification, and there are a number of benefits and challenges associated with its use [1], [2]. The 16S rRNA gene is an ideal target due to its ubiquitous presence in prokaryotic organisms and is characterized by a series of variable and conserved regions [3]. Universal PCR primers targeting different conserved regions can amplify the intermittent variable regions from a diverse selection of prokaryotes [4]. The amplified regions are subsequently sequenced allowing for genus and sometimes species level identification [5].

16S rRNA microbial identification has a number of well-documented challenges including orthologue (between organisms) and paralogue (within an organism's genome) sequence diversity [6], [7]. Another major challenge occurs due to differential microbial DNA contamination found in the laboratory or reagents, leading to erroneous results [8], [9], [10]. In addition, disparities between different laboratories lead to poor reproducibility [3], [7]. 16S rRNA gene sequencing is currently performed using both traditional Sanger sequencing and Next Generation Sequencing (NGS). Sequence read lengths, throughput, and base call accuracy vary by sequencing platform.

NGS platforms, which are increasingly being used, have relatively short reads (75 base pairs (bp) to 500 bp), but much deeper coverage (i.e., higher number of sequence reads covering each position) per run (from approximately 1 × 10^4^ to over 1 × 10^8^ reads). Sanger sequencing offers long read lengths (∼800 bp) and lower, better-characterized error rate compared to NGS [11], [12], [13]. The disadvantage of higher error rates in NGS is often mitigated by deeper coverage. Regardless of error rate all sequencing platforms have systematic errors [11], [13]. To date, there have been no comparisons between 16S rRNA sequences from single organisms obtained using multiple platforms from different laboratories that consider the diversity of 16S rRNA gene copies (paralogues).

**Fig. 1:**
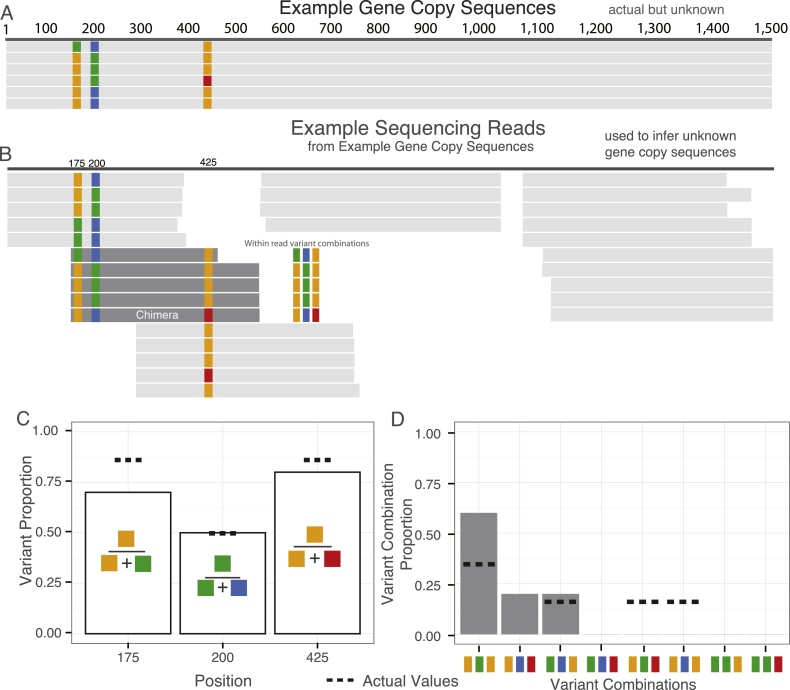
Example of the three levels of sequence analysis for multi-copy genes. An example set of six 16S rRNA gene copies with three biologically variable (colored) positions (175, 200, and 425) and example-sequencing reads are used to depict the three levels of analysis. The collection of variants, which in this example is comprised of six triplets; define the identity and provide a complete picture of the six 16S rRNA gene copies. (A) Six 16S rRNA gene copies represent the actual, but unknown, sequences within the example genome. Gray horizontal boxes stretching between 1 and 1500 bp represent individual genes; colored boxes, widened to aid visualization, indicate three variable positions. (B) Example set of “454” sequencing reads generated from the actual 16S rRNA gene copies (A) aligned to the reference. Sequencing data generated from the 16S rRNA copies can be used to make inference about the unknown gene copies from which they originated. For the first level of analysis, the identity of the biologically conserved positions, indicated in gray for both (A) and (B), is assessed using single nucleotide polymorphism calling pipelines. The second level of analysis is estimating the variant copy ratios (unknown) for the variable positions from the observed variant proportions (proportion of variants at biologically variable positions for a sequencing dataset). A statistical model was developed to estimate the variant copy ratio from the observed variant proportions. (C) The observed variant proportions, white bars, is calculated for the example read set (B), the variant proportion equation for each position is shown inside the bar. The true but unknown variant proportion (proportion of variants at biologically variable positions for a set of gene copies), calculated from the gene copies (A) is indicated with a dashed line. For the third level of analysis, the gene copy variant combination set is estimated from the sequencing data. Using a statistical model, the likely variant combination set is estimated from the observed variant combination proportions. (D) Observed variant combination proportions for reads covering the three variant positions (dark gray) in the example read set is indicated by gray bars. Dashed lines are used to indicate the true but unknown gene copy variant combination proportions. A chimeric read is included in the example read set. Chimeras are the hybrid product of two parent sequences, in this study two 16S rRNA gene copies. The combination of variants found in the chimeric read (depicted as ‘gold, blue, and red’) is not present in any of the actual 16S rRNA gene copies. The read is, therefore, the product of a chimera event between a PCR product from a gene copy with ‘gold, blue, and gold’ variant combination and a PCR product from a gene copy with a ‘gold, green, and red’ variant combination. (For interpretation of the references to color in this figure legend, the reader is referred to the web version of the article.)

The objective of this study was to compare 16S rRNA sequencing data among six international laboratories using both Sanger and NGS platforms. The newly formed Microbiology Steering Group (MBSG) of the Consultative Committee for Amount of Substance (CCQM) conducted this study (http://www.bipm.org/en/committees/cc/wg/mbsg.html). 16S rRNA sequencing data were evaluated at three levels (Fig. 1, Definitions):

(1) biologically conserved positions, for which we evaluated the nucleotide identity at each position (identical between paralogues),
(2) biologically variable positions, for which we estimated the variant copy ratio defined as the ratio of 16S rRNA gene copies featuring the two observed nucleotides at each variable position,
(3) gene copy variant combination sets, for which we estimate the set of gene copy variant combinations present in a collection of paralogous genes.

The paralogous nature of the 16S rRNA gene presents unique sequencing characteristics and challenges when considering measurement reproducibility, and its ∼1500 bp size makes it amenable to sequencing using both Sanger and NGS.

## 2 Method

### 2.1 Study overview

Participants included five national metrology institutes: National Institute for Standards and Technology (NIST, USA), LGC (UK), National Measurement Institute Australia (NMIA, AUS), National Institute of Metrology China (NIMC, CHN), Chilean Public Health Institute (ISP, Instituto de Salud Pública, CHL) and a stakeholder laboratory, American Type Culture Collection (ATCC, USA). National metrology institutes are members of the Bureau International des Poids et Mesures, the international metrology organization that manages the International System of Units (SI) as the basis for the world-wide traceability and comparability of measurement results.

Study participants sequenced the 16S rRNA gene from two certified genomic DNA reference materials from IRMM (Institute for Reference Materials and Measurements, Belgium): IRMM 449 *Escherichia coli* O157:H7 EDL933 and IRMM 447 *Listeria monocytogenes* strain 4B NCTC11994. The reference materials are certified for identity based on Sanger sequencing data for the *prfA* gene and *fliC* gene of IRMM 447 and IRMM 449, respectively [14], [15]. These two strains were selected based on their relevance to food safety, an application area of interest to the study participants.

### 2.2 Sequencing methods

**Fig. 2:**
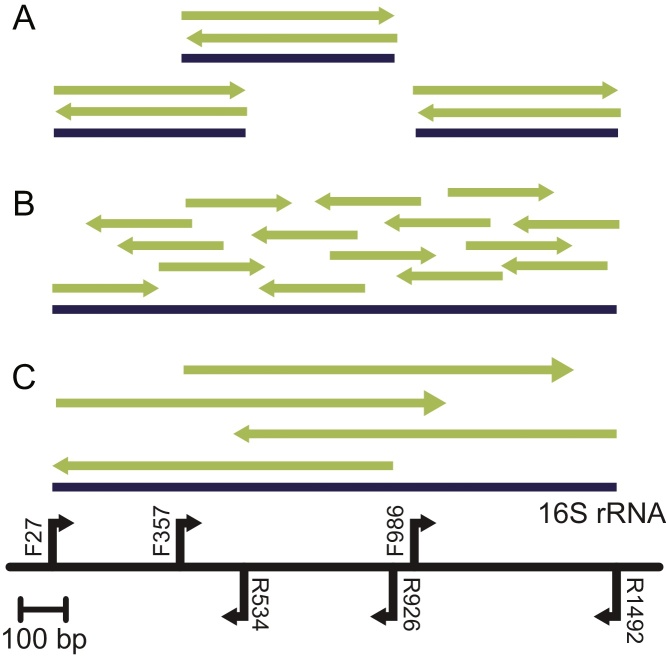
Diagram of the PCR amplicons sequenced using “454” (A), Ion Torrent (B), and Sanger sequencing (C). Blue lines represent PCR amplicons, and green arrows represent the sequencing read and direction (modified from [4]). The black line represents the 16S rRNA gene. The black arrows indicate the PCR primer direction labeled with the primer name. Primers were previously used in used by the Human Microbiome Project Jumpstart Consortium Group [3]. All PCR amplicons overlap to give full coverage of the 16 rRNA gene with the exception of “454” (A) where a 40 bp was not targeted by the PCR primers. (For interpretation of the references to color in this figure legend, the reader is referred to the web version of the article.)

Study participants sequenced the reference materials using different sequencing platforms and strategies available in their laboratories (Fig. 2, detailed protocols in Supplemental Sequencing Methods). Sequencing platforms included Sanger sequencing and two NGS platforms (Table 1 and Supplemental Results, Table 1): 454 Pyrosequencing^®^ (“454”, 454 Life Sciences, Branford, CT, USA) and Ion Torrent PGM^®^ (Life Technologies, San Francisco, CA, USA). All PCR primer sequences and thermocycler protocols were previously used by the Human Microbiome Project Jumpstart Consortium Group [3].

### 2.3 Sequence data analysis

Raw sequence data were submitted to NIST for analysis. Sequence data were compared at three levels: consensus base calls for the biologically conserved positions, variant copy ratio for positions for biologically variable positions, and the likely set of variant combinations for a collection of 16S rRNA gene copies. Raw sequence data were submitted to the GenBank SRA and Trace archives, and accession numbers for individual datasets are listed in Supplemental Sequence Methods.

The 16S rRNA gene reference sequences used during the data analysis were generated from sequences in the GenBank database (http://www.ncbi.nlm.nih.gov/genome). For *E. coli*, the individual 16S rRNA gene copy sequences were obtained from whole genome sequences for *E. coli* O157:H7 EDL933 (accession number NC_002655). *L. monocytogenes* strain 4B LL195 (accession number NC_019556) was utilized as a reference for *L. monocytogenes*, since the whole genome sequence for the reference material strain (4B NCTC11994) is not available.

All scripts and reference sequences used for data analysis are available at https://github.com/nate-d-olson/ccqm_mbwg_16S. The GitHub repository documentation includes links to a virtual image with the requirements for running the pipeline pre-installed, along with all intermediate files produced during the final run of the pipeline.

### 2.4 Biologically conserved positions

A single nucleotide polymorphism (SNP) calling pipeline was used to evaluate biologically conserved positions. To fully characterize the biologically conserved positions, results from eight pipelines (full factorial experimental design with two mapping algorithms, two mapping refinement processes, and two variant calling algorithms) were compared (Fig. SCM1). Candidate variant sites identified by the eight pipelines were evaluated for potential signs of being false positives such as strand bias. For the biologically conserved positions, datasets were compared using the following pipelines: the Torrent Mapping Alignment Program (TMAP) algorithm (https://github.com/iontorrent/TMAP) was applied to “454” and Ion Torrent datasets and the Burrows-Wheeler Aligner (BWA)-MEM algorithm (henceforth referred to as BWA, http://bio-bwa.sourceforge.net/, [16]) was applied to Sanger datasets; duplicate reads were removed for Ion Torrent datasets; realignment around indels using Genome Analysis Toolkit (http://www.broadinstitute.org/gatk [17], [18]) and the UnifiedGenotyper variant caller (part of the GenomeAnalysis Toolkit) bioinformatics pipeline were used for all datasets. The mapping files generated by the pipelines for comparing biologically conserved positions were also used for the analysis of the biologically variable positions and gene copy variant combinations. See Supplemental Computational Methods for bioinformatics pipeline specifics.

### 2.5 Estimating variant copy ratios

The datasets were compared based on the estimated variant copy ratios at the biologically variable positions. A variant copy ratio describes the ratio between the number of 16S rRNA gene copies containing each of the two nucleotides present at a given biologically variable position. Variant copy ratio estimates provide an additional level of sequence comparability. The estimated ratios were compared to the consensus variant copy ratio, defined as the estimated variant copy ratio predicted by a majority of the datasets generated in the study.

At each biologically variable position the variant copy ratio was estimated from the observed variant proportion using Bayesian statistics and binomial sampling theory. Additionally, a power analysis was performed to determine the coverage required for precise variant copy ratio predictions. See supplemental materials for detailed descriptions of the statistical methods (Supplemental Computational Methods).

### 2.6 Estimating likely variant combinations sets

Individual datasets were evaluated based on the gene copy variant combination set present in a collection of 16S rRNA gene copies (Fig. 1D). A gene copy variant combination is the combination of nucleotides at variant positions in the same copy of the 16S rRNA gene. Similar to estimating the variant copy ratios, gene copy variant combination set estimates further challenge the comparability of sequence data generated by the study participants. Read variant combination, the variant combination of individual sequencing reads for the “454” datasets and the consensus sequences from individual clones for Sanger clone libraries, was used to infer the gene copy variant combination set. Reads in the Ion Torrent datasets were too short and Sanger amplicon sequencing datasets were too small for variant combination analysis. The read variant combinations for individual “454” sequencing reads and Sanger clones were represented at the concatenated nucleotides at biologically variable positions. Observed variant combination proportions, the proportion of reads with a variant combination relative to the total number reads with variant combinations, were determined for each dataset.

A statistical analysis based on maximum likelihood was used to determine the likely variant combination set, the combinations of variants in the seven *E. coli* and six *L. monocytogenes* 16S rRNA gene copies, and described in Supplemental Computational Methods. The analysis considered the probability that the variant combinations from any individual read were the product of a chimera event. A chimera event results in a hybrid DNA sequence that is the product of two parent sequences, which for this study would be two different 16S rRNA gene copies; see Fig. 1B for an example of a chimera [19]. This analysis did not attempt to distinguish chimera events from sequencing errors occurring at biologically variable positions. In the absence of the underlying truth for the gene copy variant combinations, results from each individual dataset were compared to the values obtained using all the datasets combined.

## 3 Results and discussion

In this study we investigated the reproducibility of 16S rRNA sequencing in two microbial strains by different laboratories and platforms. All datasets concurred at biologically conserved positions, and the consensus nucleotides were identical to the reference genomes for these isolates in the GenBank database with one exception (see below). However, the precision of the results for the biologically variable positions and likely variant combinations within paralogues were dependent on the length and number of reads in the datasets (Table 1, Fig. 4, Fig. 5, and Supplemental Results).

Sequencing datasets generated by the participating laboratories varied in both read length and number of reads (Table 1). As expected [20], [21], the differences in these parameters were platform-dependent.

### 3.1 Biologically conserved positions

Biologically conserved positions were evaluated using a whole genome variant calling pipeline. Based on preliminary sequencing data, a biological variant in strain 4B NCTC11994 at position 419 not present in strain 4B LL195 was identified. The reference sequence was modified to best represent a consensus of the 4B NCTC11994 strain's 16S rRNA gene copies (paralogues). False positive SNPs, relative to the reference sequence, were identified using the eight pipelines (Table 2, Supplemental Results, Appendix Tables S4 and S5).

Regardless of the read length or number of reads in the dataset, the base calls assigned by the variant calling pipeline for the conserved positions were identical to the reference gene sequence after the 4B LL195 reference was modified as described above (Table 2, Supplemental Results, Appendix Tables S4 and S5). While the variant calling pipelines identified a number of candidate variants, upon manual inspection or application of standard filtering procedures, all were determined to be false positives. There are a number of possible causes for the false positive variant calls: (1) strand bias, due to the amplicon sequencing strategy and bioinformatics indicated by high Fisher strand-bias statistics (FS) [18]; (2) end of reads, due to low base quality at the end of sequencing reads [12]; (3) non-target region, as for the “454” datasets a 40 bp region was not targeted by the PCR primers, but was present in the dataset due to generation and sequencing of amplicons truncated by only one primer (Fig. 2); (4) homopolymer systematic sequencing error [22]; (5) end of reference, when variants were called at the last position of the reference gene due to improper read trimming (Supplemental Results, Appendix); (6) contaminants, responsible for the largest number of false positives in sequencing of *L. monocytogenes*. The contaminant reads were identified as *E. coli* using BLAST (Supplemental Results, Appendix).

### 3.2 Variant copy ratio estimate method development

The second level of sequence analysis was estimating the variant copy ratio. The variant copy ratio for the biologically variable positions was inferred from the observed variant proportions, the ratio of reads in a sequence dataset with two identified nucleotides at a biologically variable position (Fig. 1C). Traditionally, 16S rRNA sequence analysis does not take into consideration multiple paralogues [7], [23] and either consensus or a single representative sequence is used, ignoring differences between paralogues.

To estimate the variant copy ratio, the ratio of variants among 16S rRNA gene copies, an analysis based on the binomial distribution was implemented (Supplemental Computational Methods). According to binomial sampling theory, the observed variant proportions while precise (due to high coverage), differed significantly from all potential variant proportions, assuming the *E. coli* and *L. monocytogenes* strains have seven and six 16S rRNA gene copies, respectively (Supplemental Results, Figs. S1 and S2). Subsequently, given the observed variant proportions, a Bayesian approach was used to identify the most probable variant copy ratio out of the discrete set of possible ratios (Figs. S1 and S2).

The assumed 16S rRNA gene copy number is based on the number of 16S rRNA gene copies present in *E. coli* O157:H7 and *L. monocytogenes* strains with sequenced genomes in the GenBank database. The number of 16S rRNA genes was consistent among strains with sequenced genomes, increasing our confidence in this assumption. The *L. monocytogenes* strain 4b NCTC11994 had three biologically variable positions in its six 16S rRNA gene copies. The *E. coli* O157:H7 EDL933 strain had eleven variant positions in its seven 16S rRNA gene copies. The model also assumes a single organism homogenous sample and no bias in sequencing of individual gene copies.

### 3.3 Variant copy ratio estimate comparisons

The estimated variant copy ratios most frequently agreed with the consensus variant copy ratio estimates for the higher throughput datasets, such as Sanger clone libraries, “454” and Ion Torrent (Fig. 4). All variant copy ratio estimates for the “454” datasets were in agreement with the consensus ratios, and Sanger clone libraries had one disagreement. Ion Torrent datasets produced ten ratio estimates that disagreed with the consensus. All of the estimated variant copy ratios from the Sanger amplicon datasets disagreed with the consensus. This disagreement may be due to the fact that Sanger amplicon sequencing base calls, unlike Sanger clones, represent a consensus base call, which is either the majority nucleotide or an appropriate ambiguity, e.g. “N”, when the base calling algorithm cannot confidently identify a single base.

Of the ten Ion Torrent variant copy ratio estimates in disagreement with the consensus, one was for the NIMC *E. coli* dataset and nine were for the *E. coli* NIST Ion Torrent dataset. The variant copy ratio was additionally modeled without the assumption of a known copy number. Without knowledge of the copy number, the variant proportion of a given base can be any number between 0 and 1 (note that a variant copy ratio of *M:m* corresponds to variant proportion of *M*/(*M* + *m*), where *M* is the number of reads with the more abundant variant and *m* the number of reads with the less abundant variant). Using a uniform (0, 1) prior distribution, the posterior distribution for abundance proportion was computed for each dataset. Interestingly, the resulting 95% posterior credibility interval was fully contained between proportions corresponding to variant copy ratios of 6:1 and 5:2 for the NIST dataset, and did not include either. That is, both 6:1 and 5:2 are substantially displaced to either side of the average proportion from the observed data (Fig. S2).

The nine positions in disagreement for the NIST dataset are in a 36 bp region with the less abundant variant at the nine positions are on the same 16S rRNA gene copy. As only the NIST and not the NIMC Ion Torrent variant copy ratios estimates for the positions with 6:1 consensus ratios were in disagreement with the consensus (Fig. 4). The bias in variant copy ratio estimation is unlikely due a sequencing platform or library preparation method, but due to an unknown run-specific or library-specific bias.

**Fig. 3:**
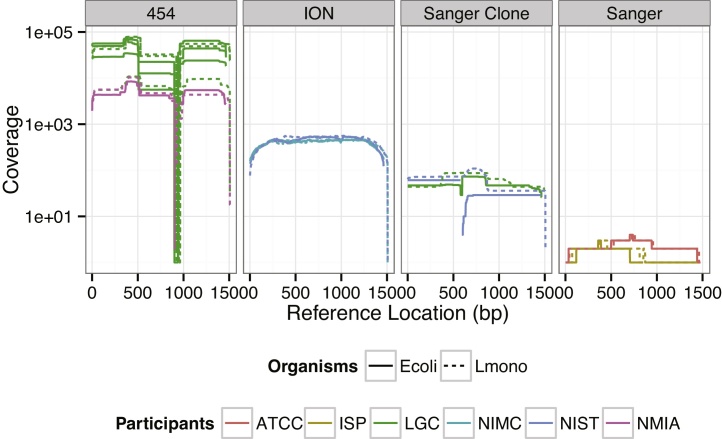
Sequencing coverage by reference base position. The depth of coverage on a log scale indicates the number of reads mapped to each position along the 16S rRNA gene reference sequence. Template organisms used to generate the datasets are indicated by solid and dashed lines for *E. coli* O157:H7 EDL 933 and *L. monocytogenes* serotype 4b strain NCTC11994, respectively.

**Fig. 4:**
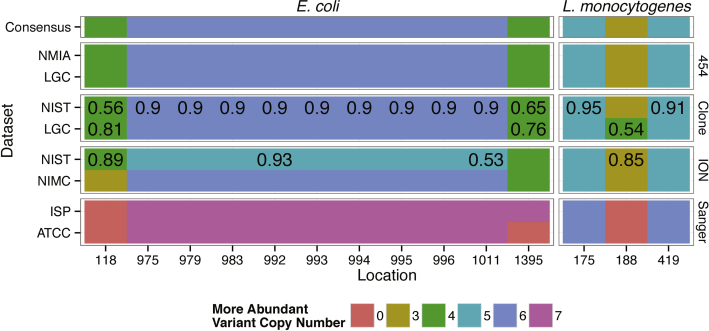
Variant copy ratio estimates for biologically variable positions. The plot is split by organism (*x*-axis) and sequencing method (*y*-axis). Location indicates the base position relative to the reference sequence. Fill color indicates the estimated number of 16S rRNA gene copies with the more abundant variant; the overall consensus more abundant variant copy number for each position is indicated in the top row of the plot. Posterior variant copy ratio estimate probabilities less than 95% are indicated.

For the Sanger clone libraries, despite having lower coverage compared to the Ion Torrent datasets (Fig. 3), the most probable variant copy ratios were in agreement with the consensus variant copy ratio estimates for all but one position (Fig. 4). However, for this position, the posterior probability of the estimated ratio was low, 0.54, and the consensus variant copy ratio fell within the 95% posterior credible interval of the variant copy ratio computed from the Sanger clone libraries (when modeled with the uniform prior distribution, as described above, Fig. S2). The Sanger amplicon-sequencing dataset contained only four reads, which was inadequate to precisely determine the variant copy ratio.

Increased sequencing coverage is required to determine the variant copy ratios for the variable position compared to the coverage required when using only the biologically conserved positions for identification. To estimate the required coverage to correctly identify the variant copy ratio with a 95% probability, a power analysis was performed. Based on the power analysis, for the 6:1 and 5:1 variant copy ratios 96 and 80 x coverage is desired, respectively, whilst for 4:3 and 3:3 variant copy ratios, 196 and 144 x coverage is required, respectively (Supplemental Computation Methods, Eq. (3)).

We also found that the mapping algorithm used to map the reads can bias the variant copy ratio estimates. When the estimated variant copy ratios were calculated from the mapping files generated by the BWA mapping algorithm for the *E. coli* positions with consensus variant copy ratios of 6:1, a majority of the variant copy ratio estimates for the NMIA “454” data were 5:2, whereas variant copy ratio estimates for the NIST Ion Torrent data were 6:1. Furthermore, analysis of characterized reference materials is required to assess the accuracy of the variant copy ratio estimates and characterize biases such as the observed mapping bias.

### 3.4 Likely variant combination set methods development

The third level of sequence analysis is a comparison of the full-length sequences for the individual gene copies (Fig. 1D). Previously only Sanger clone libraries were used to evaluate gene copy variant combination sets [7]. In the absence of such methods for NGS datasets, we developed a novel method for evaluating likely variant combination set from NGS datasets using maximum likelihood statistics and taking into consideration the rate of a chimera event occurring between biologically variant positions within 16S rRNA gene copies (Supplemental Computational Methods).

The likely variant combination sets for the two microorganisms were determined using “454” and Sanger clone library datasets. The sample size of the Sanger clone library datasets was not large enough to identify the likely variant combination set for all eleven positions for the individual 16S rRNA gene copies in *E. coli*, so the individual gene copy sequences in this organism were only determined for ten of the eleven positions. Since the true gene copy variant combination set is not known, consensus values were used for dataset comparisons. The consensus values were defined as the likely variant combination sets obtained by analyzing the combined Sanger clone library and “454” datasets using the same methods used for the individual datasets. The dataset sizes were not normalized, creating bias toward the “454” dataset due to the significantly larger number of reads in this dataset, while reducing the impact of chimeras present in the Sanger clone libraries.

### 3.5 Likely variant combination set comparisons

**Fig. 5:**
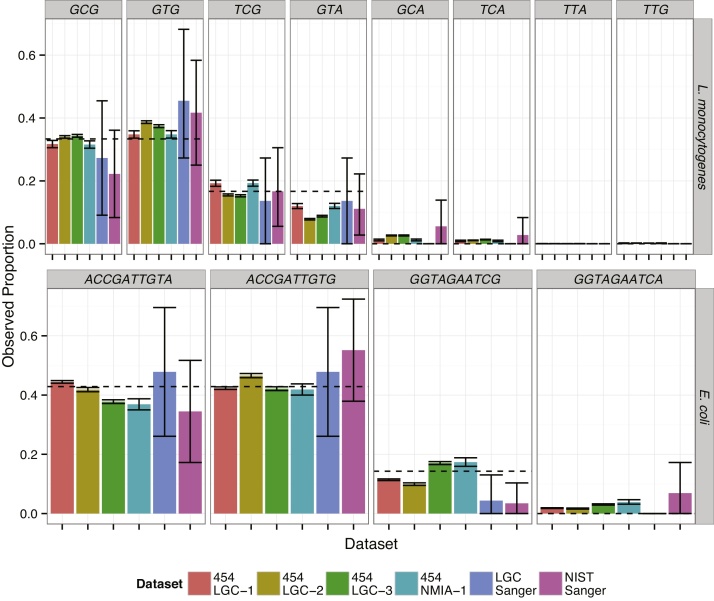
Observed variant combination set proportions. The plots are split based on the variant combination (*x*-axis) and organism (*y*-axis). The dashed line indicates the likely proportion of each variant combination determined using the four “454” datasets and two Sanger clone libraries. Error bars indicate the 95% confidence interval for the proportions calculated from the binomial distribution.

For *E. coli*, when all datasets were combined, the most likely variant combination sets was the same as the most likely variant combination sets for the individual datasets excluding two Sanger clone library datasets (Supplemental Results, Table S2). For *L. monocytogenes*, all of the most likely variant combination sets for the individual datasets were in agreement with the consensus set (Supplemental Results, Table S3). For the individual *E. coli* datasets, the likely variant combination set disagreed with the consensus set for both Sanger clone library datasets; both clone libraries had an additional copy of the gene with the variant combination “ACCGATTGTG.” The difference between the likely variant combination sets for the Sanger clone libraries and the consensus set was likely due to the small sample size (indicated by the larger 95% confidence interval) for these datasets compared to the “454” datasets (Fig. 5).

## 4 Conclusions

Utilizing our novel method for analyzing single organism 16S rRNA gene sequence data, we compared sequence data generated using multiple platforms. For the 16S rRNA bases that did not vary between gene copies (paralogues), all methods were concordant after accounting for known biases and are therefore reproducible. However, for the bases that varied between paralogues, we found that the number of reads in the dataset substantially influenced the precision of the results. To obtain method reproducibility when comparing biologically variant positions and variant combination sets, higher throughput sequencing methods such as Sanger clone libraries and NGS are required. Additionally, the choice of aligner was shown to introduce biases in the results.

The methods presented here provide additional levels of comparability between 16S rRNA gene sequences beyond comparison of the gene consensus sequence alone and can also be used for analyzing other multi-copy genes. While these methods allow for increased sequence comparability, the suitability of this method for differentiating bacteria at the strain level is unknown. With the rapid decrease in the cost of whole genome sequencing, it is unlikely that NGS sequencing of 16S rRNA will be used for strain level differentiation of individual isolates. However, the methods presented here can be applied to other multi-copy genes sequenced using NGS. The ability to determine the likely variant combination sets and estimate variant copy ratios is dependent on the size of the gene and number of paralogues. The coverage required for estimating variant copy ratios is dependent on the number of paralogues and the variant copy ratio. Additionally, the likely variant combination sets estimates are dependent on distance between variants and sequence read length.

This work represents the first interlaboratory microbial sequence comparison study for the CCQM Microbiology Steering group. Methods and protocols developed for this study will help to enable better-designed, more sophisticated future interlaboratory studies.

## Definitions

Biologically conserved positions: positions that do not vary among 16S rRNA gene copies (Fig. 1A, gray positions)
Biologically variable position: position at which nucleotides vary among 16S rRNA gene copies (Fig. 1A, colored positions)
Variant copy ratio: the ratio of variants among 16S rRNA gene copies for a biologically variable position
Variant proportion: the proportion of the more abundant variant to the total number of gene copies or reads for a biologically variable position (Fig. 1C)
Observed variant proportion: proportion of variants at biologically variable positions for a sequencing dataset
Variant copy ratio estimates: the variant copy ratios inferred from observed variant proportions
Variant combination: the combination of variants present in a single sequencing read or 16S rRNA gene copies (Fig. 1B and D)
Variant combination proportion: the proportion gene copies or reads with a given variant combination to the total number of reads with variant combinations or paralogues (Fig. 1D)
Gene copy variant combination set: the set of variant combinations present in paralogous genes
Likely set of variant combinations: the gene copy variant combination set inferred from the variant combination proportions in a sequencing dataset

## Disclaimer

Opinions expressed in this paper are the authors’ and do not necessarily reflect the policies and views of DHS, NIST, or affiliated venues. Certain commercial equipment, instruments, or materials are identified in this paper in order to specify the experimental procedure adequately. Such identification is not intended to imply recommendations or endorsement by NIST or NMIA, nor is it intended to imply that the materials or equipment identified are necessarily the best available for the purpose. Official contribution of NIST; not subject to copyrights in USA.

## Figures and Tables

**Table 1:** Summary of sequencing datasets.

Labs	Sequencing		*E. coli* read^a^		*L. monocytogenes* read^a^	
	Platform	Method	Count	Length^b^	Count	Length^b^
NIST	Sanger	Clone	92	677 (509–892)	109	848 (454–897)
	Ion	Shotgun	130,421	178 (8–337)	147,716	197 (7–344)
NMIA	“454”	Amplicon	14,817	525 (43–601)	16,273	541 (53–624)
NIMC	Ion	Shotgun	454,348	159 (8–327)	274,354	162 (8–352)
LGC	“454”	Amplicon	139,047	530 (43–773)	137,208	541 (48–692)
			62,401	529 (44–927)	127,100	543 (48–744)
			114,594	528 (43–1194)	118,995	540 (48–832)
	Sanger	Clone	94	815 (512–1106)	88	1058 (653–1178)
ATCC	Sanger	Amplicon	4	843 (745–942)	4	845 (753–934)
ISP	Sanger	Amplicon	4	498 (348–732)	4	562 (414–796)

a Raw sequence read counts and lengths reported. Appropriate read trimming was performed as part of the bioinformatics pipeline (see Supplemental Computational Methods).

b Read length in base pairs as mean (minimum–maximum).

**Table 2:** Cause of false positive variant calls for biologically conserved positions.

Organism	Plat^a^	Lab	Map^b^	Var^c^	Total	Contam^d^	End of read	End of Ref^e^	Homo-polymer	Non-target region	Strand bias
*E. coli*	454	LGC	BWA	GATK	10		8			2	
			SAMtools	2		1		1		
		TMAP	GATK	7		5			2	
			SAMtools	2		1		1		
	NMIA	TMAP	GATK	11		4			6	1
			SAMtools	4		1			3	
ION	NIMC	TMAP	SAMtools	1			1			
Sanger	NIST	TMAP	SAMtools	2			2			

*L. monocytogenes*	454	LGC	BWA	GATK	41	32	7			2	
		TMAP	GATK	8	1	4			2	1
	NMIA	BWA	GATK	1		1				
		TMAP	GATK	11		1			7	3
ION	NIST	TMAP	GATK	1						1
ION	NIST	BWA	GATK	1						1
Sanger	LGC	BWA	SAMtools	2		2				
		TMAP	SAMtools	2			2			
	NIST	BWA	GATK	1		1				
		BWA	SAMtools	2		2				
		TMAP	GATK	1		1				
		TMAP	SAMtools	3		3				
			Total	209	33	42	5	2	24	7

a Plat – sequencing platform.

b Map – mapping algorithm.

c Var – variant calling algorithm.

d Contam – contaminants.

e Ref – reference sequence that the sequence reads were mapped to.

## References

[1] Janda J.M., Abbott S.L. (2007). 16S rRNA gene sequencing for bacterial identification in the diagnostic laboratory: pluses, perils, and pitfalls. J Clin Microbiol.

[2] Tringe S.G., Hugenholtz P. (2008). A renaissance for the pioneering 16S rRNA gene. Curr Opin Microbiol.

[3] Group Jumpstart Consortium Human Microbiome Project Data Generation Working Group (2012). Evaluation of 16S rDNA-based community profiling for human microbiome research. PLOS ONE.

[4] Klindworth A., Pruesse E., Schweer T., Peplies J., Quast C., Horn M. (2012). Evaluation of general 16S ribosomal RNA gene PCR primers for classical and next-generation sequencing-based diversity studies. Nucleic Acids Res.

[5] Wang Q., Garrity G.M., Tiedje J.M., Cole J.R. (2007). Naive Bayesian classifier for rapid assignment of rRNA sequences into the new bacterial taxonomy. Appl Environ Microbiol.

[6] Fukushima M., Kakinuma K., Kawaguchi R. (2002). Phylogenetic analysis of *Salmonella*, *Shigella*, and *Escherichia coli* strains on the basis of the gyrB gene sequence. J Clin Microbiol.

[7] Pei A.Y., Oberdorf W.E., Nossa C.W., Agarwal A., Chokshi P., Gerz E.A. (2010). Diversity of 16S rRNA genes within individual prokaryotic genomes. Appl Environ Microbiol.

[8] Corless C.E., Guiver M., Borrow R., Edwards-Jones V., Kaczmarski E.B., Fox A.J. (2000). Contamination and sensitivity issues with a real-time universal 16S rRNA PCR. J Clin Microbiol.

[9] Knights D., Kuczynski J., Charlson E.S., Zaneveld J., Mozer M.C., Collman R.G. (2011). Bayesian community-wide culture-independent microbial source tracking. Nat Methods.

[10] Tanner M.A., Goebel B.M., Dojka M.A., Pace N.R. (1998). Specific ribosomal DNA sequences from diverse environmental settings correlate with experimental contaminants. Appl Environ Microbiol.

[11] Bragg L.M., Stone G., Butler M.K., Hugenholtz P., Tyson G.W. (2013). Shining a light on dark sequencing: characterising errors in Ion Torrent PGM data. PLoS Comput Biol.

[12] Ewing B., Hillier L., Wendl M.C., Green P. (1998). Base-calling of automated sequencer traces using phred. I. Accuracy assessment. Genome Res.

[13] Zook J.M., Samarov D., McDaniel J., Sen S.K., Salit M. (2012). Synthetic spike-in standards improve run-specific systematic error analysis for DNA and RNA sequencing. PLOS ONE.

[14] Van Iwaarden P., Philipp W., Emons H. (2006). EUR 22107 – DG Joint Research Centre, Institute for Reference Materials and Measurements – Certification of a Reference Material of Purified Genomic DNA from *Listeria Monocytogenes* (strain 4B, NCTC 11994) – Certified Reference Material IRMM-447.

[15] Iwaarden P.V., Philipp W., Catalani P., Prokisch J., Schimmel H., Trapmann S. (2006). EUR 22110 – DG Joint Research Centre, Institute for Reference Materials and Measurements – Certification of a Reference Material of Purified Genomic DNA from *Escherichia coli* O157 (EDL 933) – IRMM-449.

[16] Li H. (2013). Aligning sequence reads, clone sequences and assembly contigs with BWA-MEM.

[17] McKenna A., Hanna M., Banks E., Sivachenko A., Cibulskis K., Kernytsky A. (2010). The genome analysis toolkit: a MapReduce framework for analyzing next-generation DNA sequencing data. Genome Res.

[18] DePristo1 M.A., Banks E., Poplin R.E., Garimella1 K.V., Maguire J.R., Hartl C. (2011). A framework for variation discovery and genotyping using next-generation DNA sequencing data. Nat Genet.

[19] Haas B.J., Gevers D., Earl A.M., Feldgarden M., Ward D.V., Giannoukos G. (2011). Chimeric 16S rRNA sequence formation and detection in Sanger and 454-pyrosequenced PCR amplicons. Genome Res.

[20] Kircher M., Kelso J. (2010). High-throughput DNA sequencing – concepts and limitations. BioEssays: news and reviews in molecular. Cell Dev Biol.

[21] Shokralla S., Spall J.L., Gibson J.F., Hajibabaei M. (2012). Next-generation sequencing technologies for environmental DNA research. Mol Ecol.

[22] McElroy K.E., Luciani F., Thomas T. (2012). GemSIM: general, error-model based simulator of next-generation sequencing data. BMC Genomics.

[23] Větrovský T., Baldrian P. (2013). The variability of the 16S rRNA gene in bacterial genomes and its consequences for bacterial community analyses. PLOS ONE.

