# Supplementary material for: International interlaboratory study comparing single organism 16S rRNA gene sequencing data: Beyond consensus sequence comparisons
Source: Biomol Detect Quantif. 2015 Mar 5;3:17–24. doi: 10.1016/j.bdq.2015.01.004 (PMC4822220; doi:10.1016/j.bdq.2015.01.004)
Supplement: Supplementary file 1 [file mmc1.pdf]

Interlaboratory Study Participants
Sequencing Method Details

Participant: ATCC 2

Participant: ISP 3

Participant: LGC 4

Participant: NIMC 6

Participant: NIST 8

Participant: NMIA 10

Participant: ATCC

Sequencing method: Sanger Amplicon

NCBI Accession

**Trace**	***E. coli***	***L. monocytogenes***
	TI2337666699-2337666702	TI2337666695-2337666698

PCR

Primers: 27F and 1492R primers from IDT (http://www.idtdna.com)

Reaction: Molecular grade H_2O (CellGro), 10X PCR buffer (Invitrogen), 50 mM $MgCl_2$ (Invitrogen), dNTPs (USB), and Platinum Taq (Invitrogen)

Thermocycler conditions: 96°C initial 1 m; 25 cycles of 96°C for 10 s, 50°C s; 4 min at 60°C, and a 4°C hold.

Gel Electrophoresis: E-gel (Invitrogen), 100 bp Ladder (Invitrogen)

Quantification: Qubit dsDNA HS assay kit

PCR clean up: QIAquick PCR purification kit (QIAGEN)

**Equipment**	**Manufacturer**	
Thermocycler	Applied Biosystems	Verti 96 Well Thermocycler
Gel Electrophoresis	Invitrogen	EGel System

Notes:

Sequencing

Sanger

**Reagents**	**Manufacturer**	
Sequencing	Applied Biosystems	BigDye v3.1
	Genetix	halfBigDye v3
	Applied Biosystems	POP7
		10X Buffer 1 (Anode)
		10X Buffer 2 (Cathode)

Notes: Sequenced using 3500 Automatic genetic analyzers

Participant: ISP

Sequencing method: Sanger Amplicon

NCBI Accession

Trace	*E. coli*	*L. monocytogenes*
	TI2337666688-2337666690	TI2337666691-2337666694

PCR

Primers: 27F and 1492R primers

Reaction: PfuUltra II fusion HS DNA polymerase and buffer (Statagene); 5 ul buffer, 1 ul dNTPs, 1.2 ul forward and reverse primers, 0.5 ul taq polymerase, 5 ul template, and 26.5 ul water

Thermocycler conditions: 94°C for 5 min followed by 30 cycles of 94°C for 30 s, 63°C for 1 m, and 72°C for 2 m, then final extension of 72°C for 7 m.

Gel Electrophoresis: 2.0% Agrose SizeSelect EGels (Invitrogen) and 100 bp – 4 kb ladder (Invitrogen)

Quantification: Qubit dsDNA HS assay kit

PCR clean up: QIAquick PCR purification kit (QIAGEN)

Equipment	Manufacturer	
Thermocycler		G-Strom GS00482
	Life Technologies	Veriti

Notes:

Sequencing

Sanger

Reagents	Manufacturer	
Sequencing	Applied Biosystems	Kit BigDye Terminator v3.1 Cycle Sequencing Kit
		BigDye XTerminator Purificaiton Kit

Notes: Sequenced using 3500 Automatic genetic analyzers

Participant: LGC

Sequencing method: Sanger clone and "454"

NCBI Accession

SRA	*E. coli*	*L. monocytogenes*
**BioProject**	SRP032371	SRP032441
**BioSample**	SRS497310	SRS503366
**Experiment**	SRX370691	SRX378570
**Run**	SRR1020906	SRR1031054
	SRR1020910	SRR1031055
	SRR1021199	SRR1031056

Trace	*E. coli*	*L. monocytogenes*
	TI2337666496- 2337666591	TI2337666592-2337666687

PCR

Primers: Sanger clone library 27F-1492R; "454" 27F-534R, 357F-926R, and U926-1492R

Reaction: FastStart High Fidelity PCR System, dNTP pack (Roche)

Thermocycler conditions: Sanger clone library: 94 °C 3 min, 35 cycles of (95 °C 15 sec, 65 °C 45 sec, 72 °C 1 min 40 sec), 72 °C 8 min, 4 °C hold; "454": 94 °C 3 min, 35 cycles of (95 °C 15 sec, 65 °C 45 sec, 72 °C 1 min), 72 °C 8 min, 4 °C hold

Gel Electrophoresis: 1.2% Agarose Flash Gel (Lonza), 100 bp – 4kb DNA marker (Lonza), and 5X Gel loading buffer (Lonza)

Quantification: Bioanalyzer High Sensitivity DNA Kit

PCR clean up: Sanger – ethanol (Sigma) precipitation; "454" – QIAquick PCR purification system (QIAGEN) following manufacturer's instructions.

Equipment	Manufacturer	
**Thermocycler**	Bio-Rad	Tetrad DNA Engine 2
**Quantification**	Agilent	2100 Bianalyzer
**Gel Electrophoresis**	Lonza	FlashGel System

Notes: Triplicate PCRs pooled before Sanger sequencing. Triplicate PCRs run independently for "454".

Sequencing

Sanger Clone Library

**Reagents**	**Manufacturer**	
Cloning kit	Promega	pGEM-T easy
Culture	New England Biolads	5-alpha Competent *E. coli*
Purification	EMP Biotech, Berlin	Filter plates

Notes: Sanger sequencing performed by LGC Genomics with ABI3730XL, 50 cm array, POP7 and BigDye3.1. Primers pGEM vector primers, 357F and 926R

454

**Reagents**	**Manufacturer**	
Library Preparation	Agencourt	AMPure Beads
	Roche	emPCR (Lib-A) Reagents Kit
		emPCR Oil and Breaking Kit
		emPCR Bead Recovery Reagents
	Sigma	Isopropanol (100%)
	Fisher Scientific	NaOH (10N)
Sequencing reagents	Roche	Sequencing Buffer Kit
		Sequencing Reagents Kit
		Packing Beads and Supplemental CB
Quality control	Agilent	Bioanalyzer 7500 kit, 1000 reagents

**Equipment**	**Manufacturer**	
Sequencer	Roche	GS Junior
Quantification	Agilent	2100 Bioanalyzer
Thermocycer	Applied Biosystems	GeneAmp PCR System 9700
	Agilent	Stratagene MX300P

Notes:

Participant: NIMC

Sequencing method: Ion Torrent

NCBI Accession

**SRA**	***E. coli***	***L. monocytogenes***
**BioProject**	SRP032371	SRP032441
**BioSample**	SRS497311	SRS497334
**Experiment**	SRX371248	SRX371884
**Run**	SRR1021459	SRR1022527

PCR

Primers: 27F and 1492R (synthesized by Invitrogen)

Reaction: TaqMan GeneExpressionMasterMix (Life Technologies)

Thermocycler conditions: 95°C 10 min; 30 cycles of 95°C 1 min, 55°C 30 sec, 72°C 1:30; 10 min 72°C

Gel Electrophoresis:

Quantification:

PCR clean up: AMPure Beads (Agencout)

**Equipment**	**Manufacturer**	
Thermocycler	Life Technologies	Veriti 96 well Thermal Cycler
Quantification	Agilent	2100 Bianalyzer
Gel Electrophoresis	iBase	EGel

Notes:

Sequencing

Ion Torrent

**Reagents**	**Manufacturer**	
Library Preparation		
Sequencing reagents		
Quality control		

**Equipment**	**Manufacturer**	

Sequencer	Life Technologies	Ion Torrent PGM
		Ion OneTouch DL
		Ion OneTouch ES
Quantification	Agilent	2100 Bioanalyzer
Thermocycer	Life Technologies	Veriti 96 well Thermal Cycler

Notes:

Participant: NIST

Sequencing method: Sanger clone and Ion Torrent

NCBI Accession

SRA	*E. coli*	*L. monocytogenes*
**BioProject**	SRP032371	SRP032441
**BioSample**	SRS497309	SRS497333
**Experiment**	SRX370690	SRX371883
**Run**	SRR1020876	SRR1022526

Trace	*E. coli*	*L. monocytogenes*
	TI-2337665137-2337665280	TI-2337665281-2337665424

PCR

Primers: 27F and 1492R primers from Operon Eurofin MWG salt free purification

Reaction: 18 ul Platinum PCR SuperMix High Fidelity, 0.5 ul forward and reverse primer, 1 ul template

Thermocycler conditions: 95°C 5 min; 35 cycles of 95°C 15 sec, 55°C 30 sec, 68°C 1:30; 7 min 68°C

Gel Electrophoresis: 2.0% Agrose SizeSelect EGels (Invitrogen) and 100 bp – 4 kb ladder (Invitrogen)

Quantification: Qubit dsDNA HS assay kit

PCR clean up: QIAquick PCR purification kit (QIAGEN)

Equipment	Manufacturer	
Thermocycler	Bio-Rad	GeneAmp PCR System 9700
Quantification	Agilent	2100 Bianalyzer
Gel Electrophoresis	Invitrogen	EGel System

Notes: 8 replicate PCRs pooled after purification prior to sequencing.

Sequencing

Sanger Clone Library

Reagents	Manufacturer	
Cloning kit	Promega	pGEM-T easy system
Culture	Teknova Inc.	LB 100X Amp and ITPG agar plate
	Invitrogen	S.O.C. Medium

Notes: Colonies on selective agar plates sent to Functional Biosciences Incorporated for Sanger sequencing using T7, SP6, and 926R primers.

Ion Torrent

Reagents	Manufacturer	
Library Preparation	Agencourt	AMPure Beads
	Life Technologies	Ion Xpress Fragment Library Kit
		Ion Xpress Barcode Adaptor Kit
		Ion OneTouch 200 Template Kit v2 DL
Sequencing reagents		IonPGM 200 kit
		316 chip
Quality control	Agilent	Bioanalyzer 7500 kit, 1000 reagents
	Life Technologies	Ion Torrent library quantification kit
		IonSphere Quality Control Kit

Equipment	Manufacturer	
Sequencer	Life Technologies	Ion Torrent PGM
		Ion OneTouch DL
		Ion OneTouch ES
Quantification	Agilent	2100 Bioanalyzer
Thermocycer	Applied Biosystems	7500 Real Time PCR System

Notes: 1/30 of total sequencing run used for *E. coli* and *L. monocytogenes* 16S sequencing

Participant: NMIA

Sequencing method: "454"

NCBI Accession

**SRA**	***E. coli***	***L. monocytogenes***
**BioProject**	SRP032371	SRP032441
**BioSample**	SRS497312	SRS503365
**Experiment**	SRX370991	SRX378569
**Run**	SRR1021212	SRR1031053

PCR

Primers:

**Target**	**Primer name**	**Combined seq***
**UHTS1**	E-27F-fusion	cgtatcgcctccctcgcgcca tcag acgagtgcgt agagtttgatcAtggctcag
	E-534R-fusion	ctatgcgccttgccagcccgc tcag acgctcgaca attaccgcggctgctgg
**UHTS2**	E-357F-fusion	cgtatcgcctccctcgcgcca tcag agacgcactc cctacgggaggcagcag
	E-926R-fusion	ctatgcgccttgccagcccgc tcag agcactgtag ccgtcaattcAtttGagt
**UHTS3**	E-U968F-fusion	cgtatcgcctccctcgcgcca tcag atcagacacg aacgcgaagaaccttac
	E-1492R-fusion	ctatgcgccttgccagcccgc tcag atatcgcgag tacggTtaccttgttaCgactt
**UHTS1**	L-27F-fusion	cgtatcgcctccctcgcgcca tcag cgtgtctcta agagtttgatcctggctcag
	L-534R-fusion	ctatgcgccttgccagcccgc tcag ctcgcgtgtc attaccgcggctgctgg
**UHTS2**	L-357F-fusion	cgtatcgcctccctcgcgcca tcag tagtatcagc cctacgggaggcagcag
	L-926R-fusion	ctatgcgccttgccagcccgc tcag tctctatgcg ccgtcaattcCtttGag
**UHTS3**	L-U968F-fusion	cgtatcgcctccctcgcgcca tcag tgatacgtct aacgcgaagaacctta
	L-1492R-fusion	ctatgcgccttgccagcccgc tcag tactgagcta tacggCtaccttgttaCgactt

*Combined sequence primer= Primer A or B, Key, MID, template specific 5' to 3', capital letters indicate modifications to standard primers where ambiguous bases were replaced by target specific bases

Reaction: FastStart High Fidelity PCR System (Roche), dNTP Pack, IDT Fusion primers, 40 ng template

Thermocycler conditions: 95C for 5 min, followed by 35 cycles of 95°C for 30 s, 55°C for 30 s, and 72°C for 1 min, with a 5 min 72°C final extension and 4°C hold.

Gel Electrophoresis: agarose gel electrophoresis and Agilent Bioanalyzer

Quantification: Agilent Bioanalyzer (UV Spectroscopy used to estimate amount of DNA to load onto the Bioanalyzer DNA chips)

PCR clean up: AMPure Beads (Agencourt)

**Equipment**	**Manufacturer**	
Thermocycler	Bio-Rad	C1000 Touch Thermal cycler
UV Spectrophotomer	Eppendorf	Biophotometer
Gel Electrophoresis	Agilent	Bioanalyzer

Notes: Amplicons were prepared in triplicate and pooled

<u>Sequencing</u>

454

**Reagents**	**Manufacturer**	
Library Preparation	Agencourt	AMPure Beads
	Roche	Emulsion PCR Kit Lib A
		emPCR Oil and Breaking Kit
		emPCR Bead Recovery Reagents
Sequencing reagents	Roche	Sequencing Buffer Kit
		Packing Beads and Supplemental CB
Quality control	Agilent	Bioanalyzer

**Equipment**	**Manufacturer**	
Sequencer	Roche	GS Junior
Quantification	Agilent	2100 Bioanalyzer
Thermocycer	Applied Biosystems	GeneAmp PCR System 9700
	Agilent	Stratagene MX300P

Notes:
