# Supplementary material for: International interlaboratory study comparing single organism 16S rRNA gene sequencing data: Beyond consensus sequence comparisons
Source: Biomol Detect Quantif. 2015 Mar 5;3:17–24. doi: 10.1016/j.bdq.2015.01.004 (PMC4822220; doi:10.1016/j.bdq.2015.01.004)
Supplement: Supplementary file 2 [file mmc2.pdf]

Supplemental Computational Methods

Sequence processing

Raw sequence data were submitted by each participating laboratory to NIST for analysis. All scripts and required reference files needed to re-run the analysis presented in this manuscript are available in the github repository at http://github.com/nate-d-olson/ccqm_mbwg_16S. Figure SC1 is a diagram of the bioinformatics pipeline.

For Sanger sequencing, data were submitted to NIST in the form of chromatograms. For Ion Torrent and "454" sequences, data were submitted as sff or fastq files. The sff files were demultiplexed using mothur version 1.32.1 (www.mothur.org). Sanger reads were trimmed based on base quality and vector sequences were removed using Geneious R7.1 (www.geneious.com). Raw filtered Sanger fastq files are included in the github repository. For the Sanger clone libraries, the reads for individual clones were assembled in Geneious R7.1 and exported as bam file; these are also included in the github repository.

Identification of variants in biologically conserved positions

A single nucleotide polymorphism (SNP) calling pipeline was used to evaluate biologically conserved positions. To validate the bioinformatics pipeline, eight pipelines were evaluated (Fig SC1, executed with *ccqm_pipeline_comparison.sh*). The eight pipelines were a full-factorial combination of two mapping algorithms, two mapping refinement procedures, and two variant calling algorithms. The two mapping algorithms used were Torrent Mapping Alignment Program (TMAP) version 3.4.1 (http://github.com/iontorrent/tmap) and Burrows-Wheeler Aligner (BWA) version 0.7.9a-r786 "mem" algorithm (further referred to as BWA) [http://bio-bwa.sourceforge.net/,1], with and without realignment around indels [GATK, GenomeAnalysisTK-2.3-9, http://www.broadinstitute.org/gatk/,2,3] and duplicate read removal (picard-tools-1.115, http://picard.sourceforge.net/, only for Ion Torrent datasets). The two variant callers were the GATK UnifiedGenotyper [2] and SAMtools mpileup (sam) [version 0.1.18, http://samtools.sourceforge.net/,4]. Datasets were compared using the following pipelines: TMAP for "454" and Ion Torrent datasets and BWA for Sanger datasets, duplicate reads were removed for Ion Torrent datasets, realignment around indels was performed for all datasets, and the UnifiedGenotyper variant caller was used for all datasets bioinformatics pipeline. The same pipelines used to compare the biologically conserved positions were also used to for the variant copy ratio and variant combination estimates excluding the use of the UnifiedGenotyper variant caller.

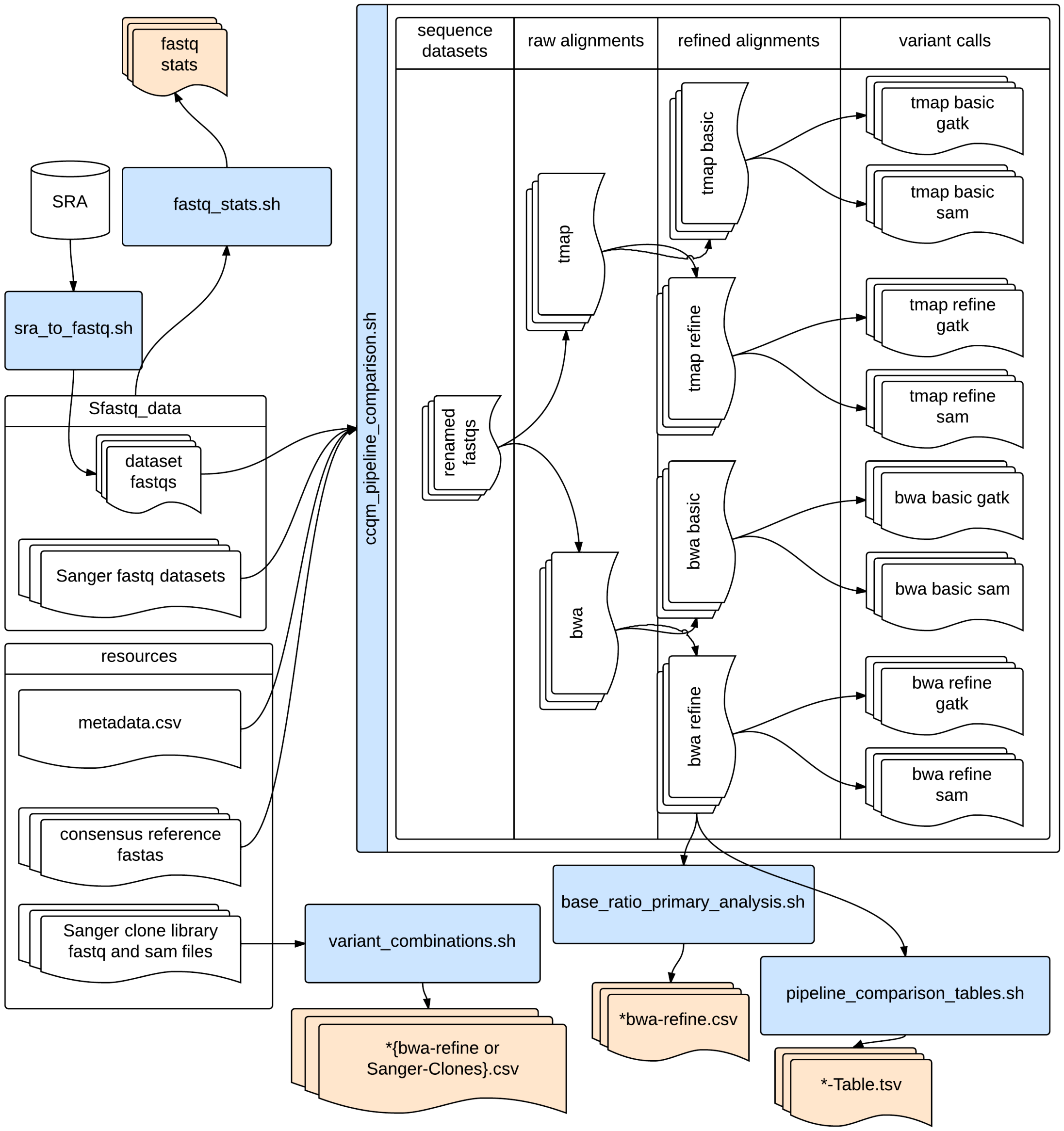

Fig SCM1: Diagram of the bioinformatics pipeline. Output files used in downstream statistical analysis are indicated in brown and scripts are indicated in blue. Note the full-factorial design for the pipeline comparisons, resulting in eight sets of variant call for each datasets.

Statistical analysis pipeline

The statistical analysis pipeline consisted of four main components: summary of sequence datasets, dataset summary statistics, analysis of biologically variable positions and variant string analysis (Fig SCM2).

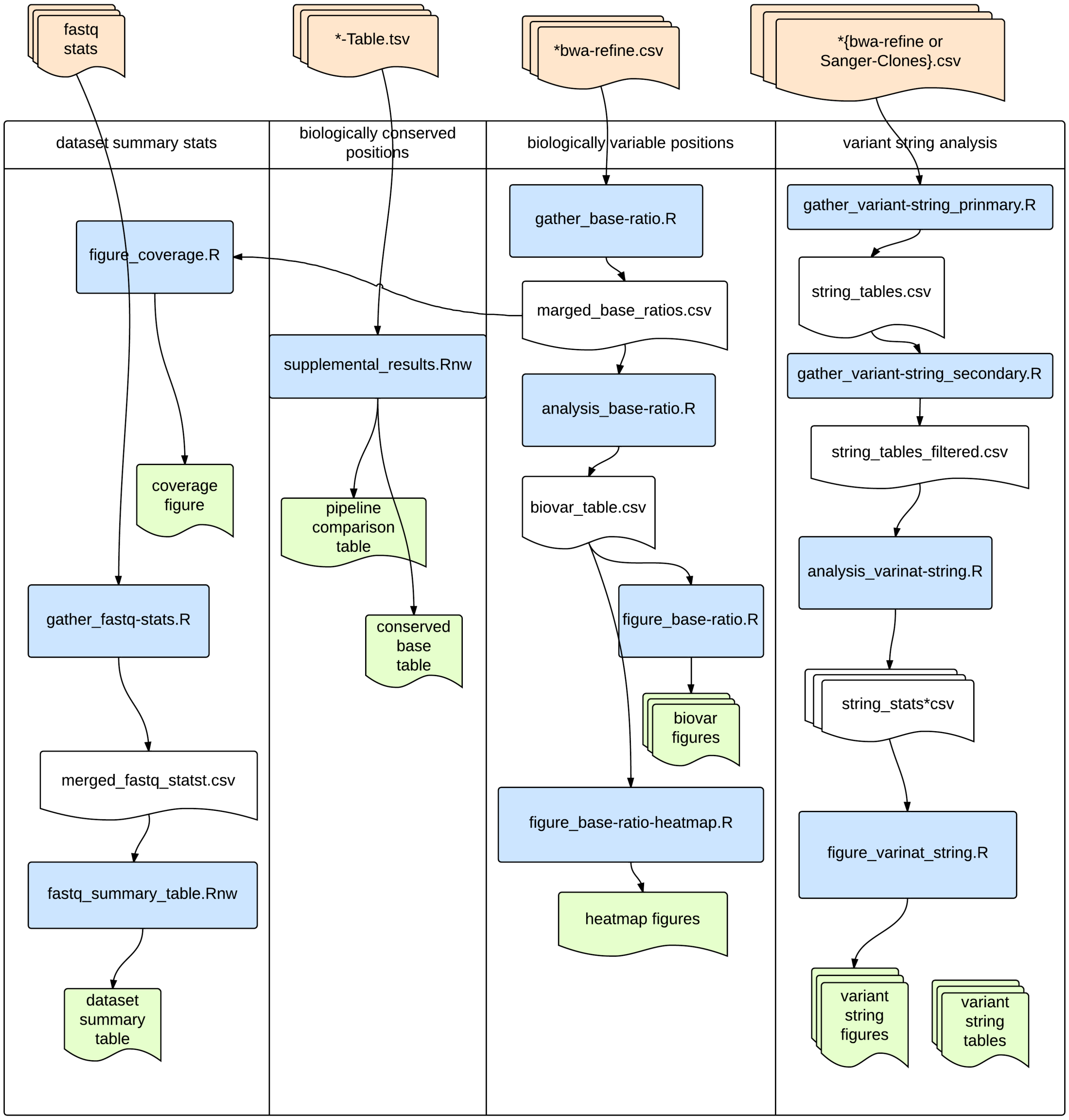

Fig SCM2: Diagram of the statistical analysis pipeline. Input files used from the bioinformatics pipeline are indicated in brown and scripts are in blue the output tables and figures are indicated in green. The variant string table was generated as part of the supplemental results using the *supplemental_results.Rnw* script.

Predicting variant copy ratios for biologically variable positions

The predicted variant copy ratio is the predicted number of copies for the two variants at each of the biologically variable positions. For example, the potential variant copy ratios for *L. monocytogenes* are 6:0, 5:1, 4:2, 3:3, 2:4, 1:5 and 0:6. Let $\mathrm{M{:}\,m}$ denote the variant copy ratio for a given variable position, where M is the more abundant variant and m is the less abundant variant. The binomial distribution (Eq. 1) was used to calculate $l(\mathrm{M{:}\,m}\ |\mathrm{y,c})$, the likelihood that the true variant copy ratio is $\mathrm{M{:}\,m}$ given that the ratio of variants in reads containing the specified variable position

is $y: (c-y)$, where y denotes the number of reads containing major variant and c denotes coverage (i.e. total number of reads overlapping specified variable position).

Equation 1: Likelihood of variant copy ratio $M{:}\,m$ given number of reads containing major variant (y) and coverage (i.e. total number of reads overlapping specified variable position) (c).

$$l(M{:}\,m\,|y,c) = \binom{c}{y} p^y (1-p)^{c-y},$$

where $p = \frac{M}{M+m}$.

From the resulting set of likelihoods, Bayes' theorem was used to assign posterior probabilities to each variant copy ratio. Assuming *a priori* that all potential variant copy ratios are equally likely, the posterior probability for a variant copy ratio is the likelihood of that variant copy ratio ($l(M{:}\,m\,|y,c)$) normalized by the sum of the likelihoods for all possible variant copy ratios (Eq. 2).

Equation 2: Posterior Probability of variant copy ratios given reads and total number of gene copies (n)

$$\Pr(M{:}\,m|n,y,c) = \frac{l(M{:}\,m\,|y,c)}{\sum_{M'{:}m' = 0{:}n,1{:}(n-1),\ldots,n{:}0} l(M'{:}\,m'\,|y,c)}$$

The required coverage for the different variant copy ratios was calculated using a power analysis. Specifically, we determined the coverage required so that the true variant copy ratio would be correctly identified as the most likely copy ratio given the data with probability 0.95. This is equivalent to determining the coverage required so that 95% of the sampling distribution for the proportion of reads with the more abundant variant $\left(\frac{y}{c}\right)$ is contained within the interval $\left(\frac{M-0.5}{M+m}, \frac{M+0.5}{M+m}\right)$. For large coverages, the sampling distribution for the proportion of reads with the more abundant variant $\left(\frac{y}{c}\right)$ can be approximated as a normal distribution with mean $\frac{M}{M+m}$ and variance $\frac{p(1-p)}{c}$, where $p = \frac{M}{M+m}$. The central 95% of this distribution will lie within $\left(\frac{M-0.5}{M+m}, \frac{M+0.5}{M+m}\right)$ when $1.96\sqrt{\frac{p(1-p)}{c}} \leq \frac{0.5}{n}$, where $n = M + m$ is the total copy number. Solving this inequality for c yields $c \geq 4 \times 1.96^2 \times M \times m$. We simplify this expression and use the following equation (Eq. 3) for estimating desired coverage:

Equation 3: Desired Coverage Estimates

$$Desired\ Coverage = 16 \times M \times m$$

Individual gene copy variant combinations maximum likelihood statistics calculation

Here we describe the method used to calculate the likely variant combination set for 16S rRNA paralogues or gene copies, using *L. monocytogenes* as an example. *L. monocytogenes* has three biologically variant positions, each of which having two possible nucleotides. There are thus $2^3=8$ possible variant combinations, referred to as variants strings. For each possible variant string, we estimate the number of gene copies containing the given variant string using a maximum likelihood approach described below following a diagram to illustrate our approach.

Chimeras play an important role when estimating variant combination presence in gene copies from a set of reads. As a general concept, if no chimeras occur along a read, then the read must exactly match one of the gene copies actually present in the organism. When a chimera occurs, however, read fragments separated by a chimera are assumed to be independent draws of the corresponding fragments from the gene copy population. Figure SC3 provides an illustrative example for a gene with three biological variant positions: A, B and C (see Fig. SC3.A). The four columns comprising Fig. SC3.E reflect the four possible chimera combinations: "No Chi" applies when no chimera occurs either between positions A and C; "Chi 1" applies when at least one chimera occurs between positions A and B and no chimera occurs between positions B and C; "Chi 2" applies when no chimera occurs between positions A and B and at least one chimera occurs between positions B and C; "Chi 1 & Chi 2" applies when at least one chimera occurs between positions A and B and at least one chimera occurs between positions B and C. Each of the 8 variant combinations are repeated in each of the four columns, where 0 and 1 respectively represent the minor and major base for each variable positions (A, B, and C). Within each column, the individual bases are grouped according to their respective chimera structures.

In this example, suppose a read corresponding to variant combination 1 from Fig. SC3.C, in which all three biologically variant positions have their major base (i.e. A=B=C=1), is observed. Read fragments that match the corresponding fragment from variant combination 1 are enclosed in green rectangles, while read fragments that do not match are enclosed in blue rectangles. If the read contains no chimeras, then it must have originated from a gene copy with variant combination 1. If a chimera occurs between positions A and B, then the base at variable position A could have come from any of variant combinations 1-4, and the read fragment containing positions B and C could have come from either variant combination 1 or 5. Similarly, if a chimera occurs between positions B and C, then the read fragment containing positions A and B could have come from either variant combination 1 or 2 and the base at variable position C could have come from any of variant combinations 1, 3, 5, or 7. If chimeras occur both between variable positions A and B as well as between positions B and C, then the base at position A could have come from any of variant combinations 1-4, the base at position B could have come from any of variant combinations 1, 2, 5, or 6, and the base at position C could have come from any of variant combinations 1, 3, 5, or 7. A generalized and more technical description follows.

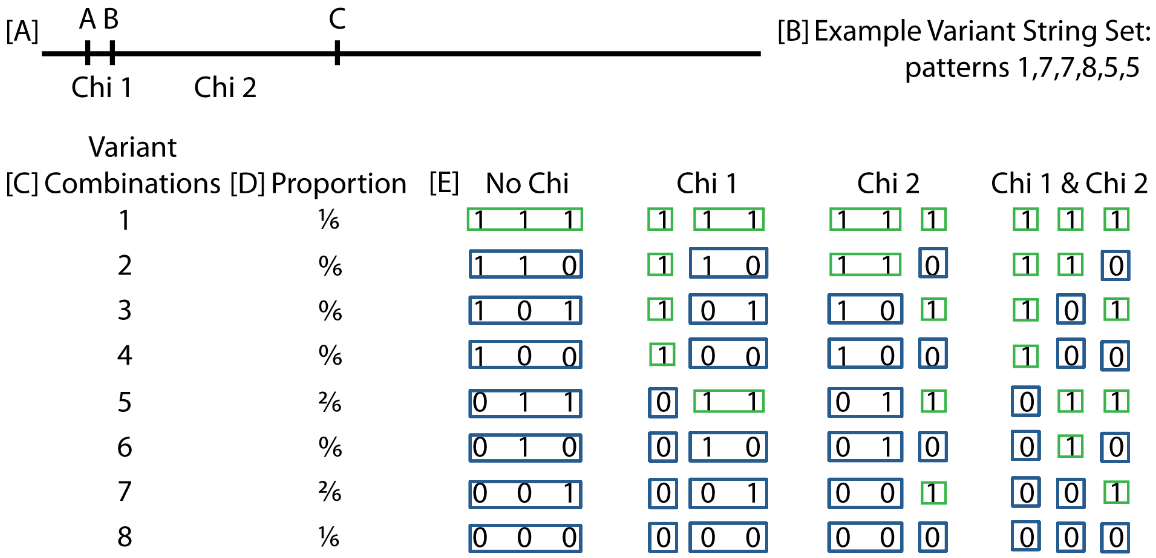

Figure SC3. Diagram of the method used for the maximum likelihood gene copy variant combination calculations. [A] Represents the 16S rRNA gene sequence and the relative positions of the three biologically variable bases (A, B, and C). With Chi 1 and Chi 2 representing the distance in base pairs between variable positions A – B and B – C respectively. [B] Provides an example gene copy set, where the numbers refer to the index of the potential variant combinations [C]. In this example there are six total gene copies: one copy each of variant combinations 1 and 8, and two copies each of variant combinations 5 and 7. The column labeled [C] provides indexes for variant combinations. The numbers 1-8 represent the 8 potential combinations assuming two bases at each of the three biologically variable positions (2^3 = 8). The column labeled [D] provides the expected proportion of reads originating from each variant combination, given the example set of gene copies provided in [B]. The four columns comprising [E] display the possible grouping structures, as determined by chimera occurrences.

Let $i = 1, \dots, I$ serve as an index of variant combinations (i.e. I total possible variant strings assuming only two potential bases at each position, Fig SC3.C). Let variant string i be described as an M-element vector $\overrightarrow{A_i} = \langle A_{i1} \dots A_{iM} \rangle$, where $m = 1, \dots, M$ is an index of biologically variant positions (e.g. Fig 3SC.A, positions A, B, and C). Specifically, let A_{im} indicate the presence of the more (A_{im}=1) or less abundant (A_{im}=0) base, respectively, at biologically variant position m in variant combination i.

Let the set of variant combinations actually present in the organism (e.g. Fig SC3.B) be described by the I -element vector $\vec{c} = \langle c_1 \dots c_I \rangle$, where c_i denotes the number of gene copies in the given organism that contain variant string *i*. Note that $\vec{c}$ is subject to the constraint $\sum_{i=1}^{I} c_i = C$, where *C* is total gene copy number (six for Fig SC3 and *L. monocytogenes*). Similarly, we can summarize the observed data as $\vec{Y} = \langle Y_1 \dots Y_I \rangle$, where Y_i denotes the number of observed reads with variant combination *i* (such that $\sum_{i=1}^{I} Y_i = N$, where N is total number of reads in a dataset).

Our goal is to estimate the set of variant combinations in the gene copies actually present in the organism, $\vec{c}$, from the observed data, $\vec{Y}$.

Estimating $\vec{c}$ from $\vec{Y}$ requires a model for the probability or proportion of reads that will match each variant combination. To more accurately model the probability we developed a model that takes into consideration the possibility that sequencing read in a chimera, or a PCR artifact where the sequence is the product of two gene copies. In the absence of chimeras (Fig SC3.E No Chi), this is straightforward as $\Pr(\overrightarrow{A_i}|\vec{c}) = c_i/C$. The existence of chimeras complicates this relationship. For instance, chimeras make it possible to observe reads that do not correspond to any variant string actually present in the organism.

To model the impact of chimeras, we assume that chimera occurrences are independent of one another and are equally likely to occur between any pair of immediately adjacent bases. Let p_{chi} denote the probability of a chimera occurring between a single pair of adjacent bases. We will eventually estimate p_{chi}, in addition to $\vec{c}$, from the observed data, $\vec{Y}$. Under the stated assumptions, the probability that no chimeras occur within a distance of B bases is $(1 - p_{chi})^B$ and the probability of at least one chimera occurring within a distance of B bases is $1 - (1 - p_{chi})^B$. Let the set of chimera occurrences be summarized by $\overrightarrow{Chi} = \langle Chi_1 \dots Chi_{M-1} \rangle$, where Chi_m is binary variable indicating whether at least one chimera ($Chi_m = 1$) or no chimeras ($Chi_m = 0$) occur between biologically variant positions m and m+1, and M is the number of biologically variant positions. Given the chimera rate, p_{chi}, the probability for a given chimera set $\overrightarrow{Chi}$ is then

$$\Pr(\overrightarrow{Chi}|p_{chi}) = \prod_{m=1}^{M-1} \left[\Pr(Chi_m|p_{chi})^{Chi_m} \left(1 - \Pr(Chi_m|p_{chi})\right)^{1-Chi_m}\right],$$

where $\Pr(Chi_m|p_{chi}) = 1 - (1 - p_{chi})^{dist_m}$ is the probability of at least one chimera occurring between biologically variant positions m and $m + 1$, which are $dist_m$ bases apart from each other.

The chimera set $\overrightarrow{Chi}$ describes the fragmentation of a read into sections of reads that must have originated from the same gene copy. If no chimeras occur along a read, then the read must exactly match one of the gene copies actually present in the organism. When a chimera occurs, however, read fragments separated by a chimera are assumed to be independent draws of the corresponding fragments from the gene copy population. We now illustrate the probabilities of observing variant string patterns conditional on chimera structure, $\overrightarrow{Chi}$, and gene copy set, $\vec{c}$.

Suppose a gene has three biologically variant positions and consider the probability of observing variant string i, with $\overrightarrow{A_i} = \langle A_{i1}\ A_{i2}\ A_{i3} \rangle$. If no chimera occurs within the given read, then probability of $\overrightarrow{A_i}$ is given by the fraction of gene copies exactly matching $\overrightarrow{A_i}$ (i.e. $\Pr(\overrightarrow{A_i}|\overrightarrow{Chi} = \langle 0\ \ 0 \rangle, \vec{c}) = c_i/C$).

If at least one chimera occurs between biologically variant positions 1 and 2 and no chimera occurs between positions 2 and 3, then the probability of observing $\overrightarrow{A_\iota}$ is product of the probabilities of the partial variant strings from either side of the chimera. That is,
$\Pr\left(\overrightarrow{A_\iota}|\overrightarrow{Chi} = \langle 1\ \ 0 \rangle, \vec{c}\right) = \Pr(A_{i1}|\vec{c})\Pr(A_{i2}\ \ A_{i3}|\vec{c})$ where
$\Pr(A_{i1}|\vec{c}) = \frac{1}{C}\sum_{j:\,A_{i1}=A_{j1}} c_j$ is the fraction of gene copies whose first biologically variant position matches that of the considered variant string and
$\Pr(A_{i2}\ \ A_{i3}|\vec{c}) = \frac{1}{C}\sum_{j:\,\langle A_{i2}\ \ A_{i3}\rangle = \langle A_{j2}\ \ A_{j3}\rangle} c_j$ is the fraction of gene copies whose second and third biologically variant positions match those of the considered variant string. Similarly, if no chimera occurs between biologically variant positions 1 and 2 and at least one chimera occurs between positions 2 and 3, we have $\Pr\left(\overrightarrow{A_\iota}|\overrightarrow{Chi} = \langle 0\ 1 \rangle, \vec{c}\right) = \Pr(A_{i1}\ A_{i2}|\vec{c})\Pr(A_{i3}|\vec{c})$. Finally, if at least one chimera occurs between biologically variant positions 1 and 2 and at least one chimera occurs between positions 2 and 3, we have $\Pr\left(\overrightarrow{A_\iota}|\overrightarrow{Chi} = \langle 1\ 1 \rangle, \vec{c}\right) = \Pr(A_{i1}|\vec{c})\Pr(A_{i2}|\vec{c})\Pr(A_{i3}|\vec{c})$.

The conditional probabilities described above can be combined to provide the probabilities of observing variant string patterns conditional on chimera rate, p_{chi}, and gene copy set, $\vec{c}$. Specifically, the variant string pattern probabilities conditional on a given chimera set, $\Pr\left(\overrightarrow{A_\iota}|\overrightarrow{Chi}, \vec{c}\right)$, are weighted by the probability for the chimera set given the chimera rate, $\Pr\left(\overrightarrow{Chi}|p_{chi}\right)$, and summed across all possible chimera sets to obtain

$$\Pr\left(\overrightarrow{A_\iota}|p_{chi}, \vec{c}\right) = \sum_{\overrightarrow{Chi}} \Pr\left(\overrightarrow{A_\iota}|\overrightarrow{Chi}, \vec{c}\right)\Pr\left(\overrightarrow{Chi}|p_{chi}\right).$$

The likelihood for the observed data is then

$$\Pr\left(\vec{Y}|\ p_{chi}, \vec{c}\right) = \binom{N}{\vec{Y}}\prod_{i=1}^{n}\Pr\left(\overrightarrow{A_i}|\ p_{chi}, \vec{c}\right)^{Y_i},$$

which is maximized to produce estimates of p_{chi} and $\vec{c}$.

References

1. Li H, Durbin R (2010) Fast and accurate long-read alignment with Burrows-Wheeler transform. Bioinformatics (Oxford, England) 26: 589-595.
2. DePristo1 MA, Banks E, Poplin RE, Garimella1 KV, Maguire JR, et al. (2011) A framework for variation discovery and genotyping using next-generation DNA sequencing data. Nature genetics 43: 491-498.
3. McKenna A, Hanna M, Banks E, Sivachenko A, Cibulskis K, et al. (2010) The Genome Analysis Toolkit: a MapReduce framework for analyzing next-generation DNA sequencing data. Genome research 20: 1297-1303.
4. Li H, Handsaker B, Wysoker A, Fennell T, Ruan J, et al. (2009) The Sequence Alignment/Map format and SAMtools. Bioinformatics (Oxford, England) 25: 2078-2079.
