# Supplementary material for: International interlaboratory study comparing single organism 16S rRNA gene sequencing data: Beyond consensus sequence comparisons
Source: Biomol Detect Quantif. 2015 Mar 5;3:17–24. doi: 10.1016/j.bdq.2015.01.004 (PMC4822220; doi:10.1016/j.bdq.2015.01.004)
Supplement: Supplementary file 3 [file mmc3.pdf]

CCQM Microbial Identity 16S rRNA Interlaboratory Study

Supplemental Results

Nathan Olson

January 20, 2015

Contents

1 Biologically Conserved Positions **5**

2 Biologically Variable Positions **6**

3 Likely sets of variant combinations **9**

4 Appendix **10**

List of Figures

S1 *L. monocytogenes* observed variant proportions 7

S2 *E. coli* observed variant proportions 8

List of Tables

S1 Biological conserved base quality statistics 5
S2 *E. coli* variant combinations 9
S3 *L. monocytogenes* variant combinations 9
S4 *E. coli* positions pipeline comparison 10
S5 *L. monocytogenes* positions pipeline comparison 11

1 Biologically Conserved Positions

None of the variants for the biologically conserved positions were called using both variant callers, indicating that the variants were potential false positives (Manuscript Table 2, Tables S4 and S5). Consensus base quality statistics for biologically conserved positions are summarized below (Table S1).

Table S1: **Biologically Conserved Position Base Qualities** Characteristics of consensus based calls for conserved bases. Normalized quality values were obtained by dividing raw quality score (Raw Qual) assigned by GATK for each biologically conserved base position by the depth of coverage for that position

Org	Plat	Lab	Rep	Raw Qual	Normalized	Min	Max
Ecoli	454	LGC	1	140738.23	2.85	1.25	3.00
Ecoli	454	LGC	2	68081.73	2.85	0.63	2.98
Ecoli	454	LGC	3	128788.23	2.93	1.16	2.99
Ecoli	454	NMIA	1	11457.23	2.51	0.31	2.97
Ecoli	ION	NIMC	1	1165.23	2.78	0.59	3.14
Ecoli	ION	NIST	1	1112.23	2.48	0.51	3.16
Ecoli	Sanger	ATCC	1	34.23	17.11	9.31	31.24
Ecoli	Sanger	ISP	1	31.24	31.23	-10.00	31.24
Ecoli	Sanger	LGC	1	169.23	3.60	0.51	3.97
Ecoli	Sanger	NIST	1	115.23	3.97	-1.43	10.06
Lmono	454	LGC	1	115365.73	2.89	1.43	3.00
Lmono	454	LGC	2	103741.23	2.87	1.44	3.00
Lmono	454	LGC	3	125648.73	2.85	1.66	2.99
Lmono	454	NMIA	1	11635.23	2.41	0.79	2.92
Lmono	ION	NIMC	1	1173.23	2.81	0.33	3.14
Lmono	ION	NIST	1	1265.23	2.56	0.23	2.90
Lmono	Sanger	ATCC	1	34.23	17.11	-10.00	31.24
Lmono	Sanger	ISP	1	34.23	17.11	-10.00	31.24
Lmono	Sanger	LGC	1	169.23	3.45	1.26	3.71
Lmono	Sanger	NIST	1	242.23	3.41	2.18	3.78

All variant calls for the biologically conserved positions were evaluated for being potential false positives (Tables S4 and S5). The potential variants identified by the eight variant calling pipelines were analyzed for potential reasons for a false positive variant call. The Fisher strand bias statistic was used to classify false positive variants due to strand bias (FS <60). Variants present in non-target regions and at the end of the reference sequence were identified based on positions relative to the reference. False positive variants due to homopolymer systemic sequencing errors and a high proportion of bases covering the identified variant position were identifed by visually inspecting the mapping file. Visual inspection of the mapping files revealed a small proportion of highly similar reads that were responsible for a number of variant calls, comparison to the Genbank database using BLAST (Supplemental Results Appendix - BLAST Results) indicated the reads were the product of *E. coli* contamination in the *L. monocytogenes* LGC “454” dataset. Note that for the NIST Ion Torrent *L. monocytogenes* dataset at position 792 a variant was called by the UnifiedGenotyper Variant Calling Algorithm when the reads were mapped using both BWA and TMAP, but the FS score was only above 60 when the reads were mapped with tmap. Upon manual inspection of the results we attributed the false positive to a strand bias.

2 Biologically Variable Positions

To determine the variant copy ratios, a novel Bayesian analysis based on binomial sampling theory was developed (Supplemental Computational Methods). According to the binomial distribution, the observed variant ratios, while precise (due to high coverage), differed significantly from all potential variant copy ratios. A Bayesian approach was used to identify the most probable variant copy ratio out of the possible ratios assuming *E. coli* and *L. monocytogenes* have seven and six 16S rRNA gene copies respectively (Figs. S1 and S2).

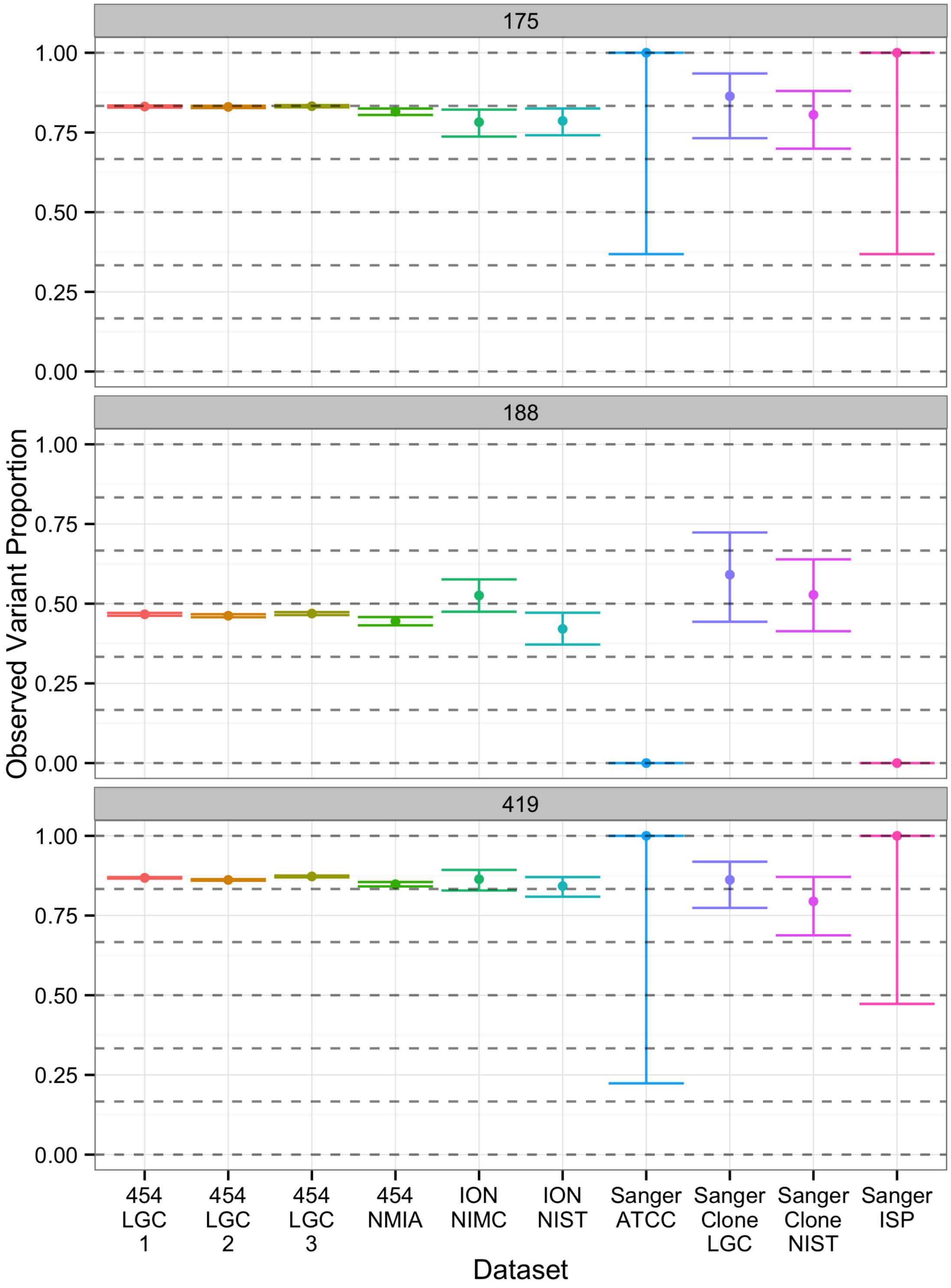

Figure S1: Observed variant proportions at three biologically variable positions (175, 188 and 419) in *L. monocytogenes*. Variable positions shown in grey box above each graph. Error bars represent the 95 % posterior credibility interval estimated from a beta binomial distribution where α is the more abundant nucleotide count + 1 and β is the less abundant nucleotide count + 1. One sided credibile intervals were calcualted for prior probabilities of 0 and 1. Grey dashed lines indicate the potential varinat copy ratios assuming six gene copies (i.e. 0:6 corresponds to 0, 2:4 to 0.33, 3:3 to 0.5, 4:2 to 0.66, 5:1 to 0.83 and 6:0 to 1).

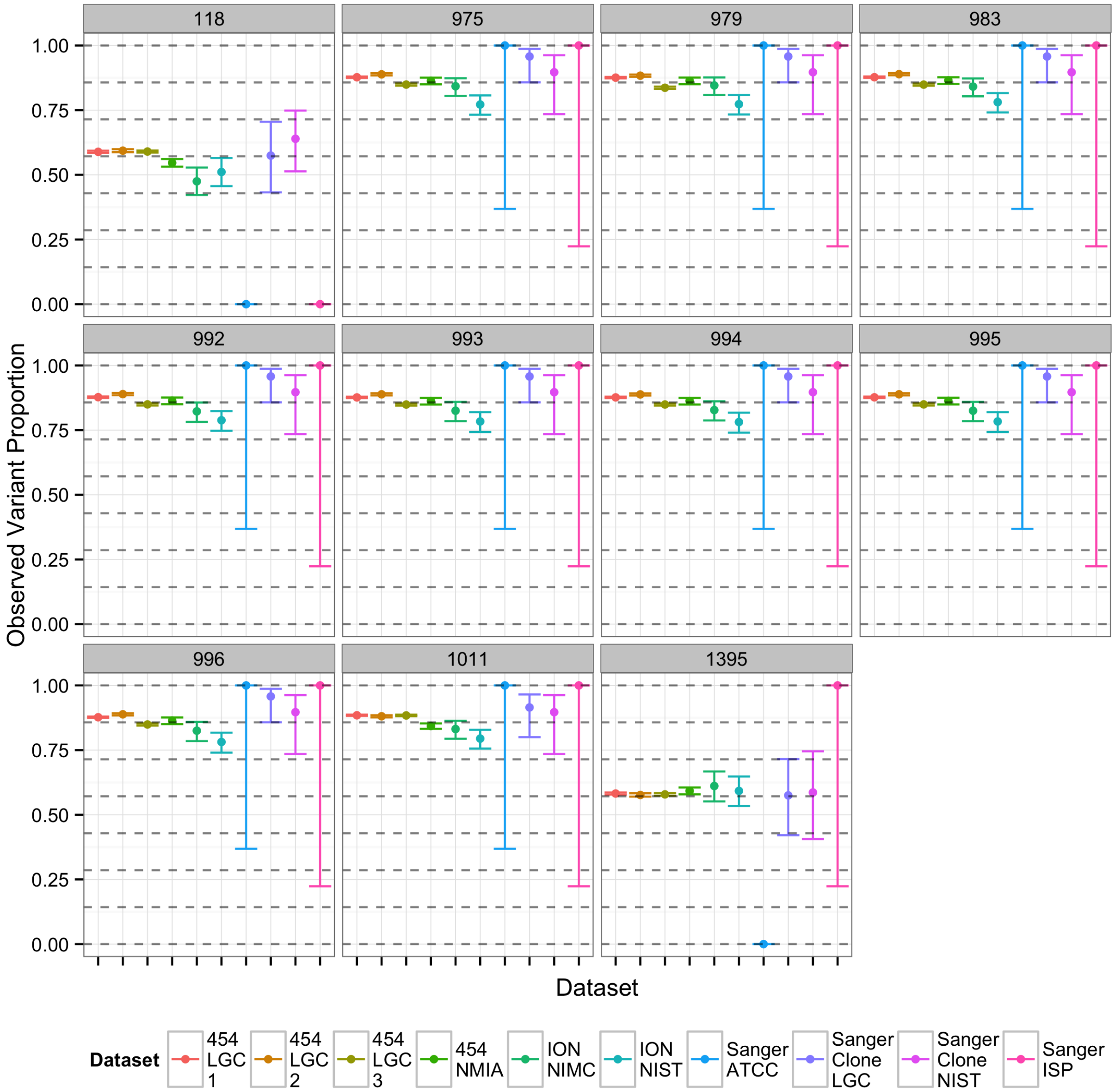

Figure S2: Observed variant proportions at eleven biologically variable positions in *E. coli*. Variable positions shown in grey box above each graph. Error bars represent the 95 % posterior credibility interval estimated from a beta binomial distribution where α is the more abundant nucleotide count + 1 and β is the less abundant nucleotide count + 1. One sided credibile intervals were calcualted for prior probabilities of 0 and 1. Grey dashed lines indicate the potential variant ratios assuming seven gene copies, (i.e. 0:7 corresponds to 0; 1:6 to 0.14; 2:5 to 0.26; 3:4 to 0.43, 4:3 to 0.57; 5:2 to 0.71; 6:1 to 0.86; and 7:0 to 1).

3 Likely sets of variant combinations

Most likely combination of variant strings for "454" and Sanger Clone library datasets (Table S2 and Table S3).

Table S2: **Estimated most likely set of variant combinations for *E. coli*.** See supplemental computation methods for how chimera and likelihood were calculated.

dataset	likelihood	chimera	ACCGATTGTA	ACCGATTGTG	GGTAGAATCA
Ecoli-454-LGC-1	0.04	275.55	3	3	1
Ecoli-454-LGC-2	0.03	275.27	3	3	1
Ecoli-454-LGC-3	0.04	242.13	3	3	1
Ecoli-454-NMIA-1	0.06	30.74	3	3	1
Ecoli-LGC-Sanger-Clones	0.05	3.55	3	4	0
Ecoli-NIST-Sanger-Clones	0.12	4.54	3	4	0
Consensus	0.04	717.62	3	3	1

Table S3: **Estimated most likely set of variant combinations for *L. monocyotogenes*.** See supplemental computation methods for how chimera and likelihood were calculated.

dataset	likelihood	chimera	GCG	GTA	GTG	TCG
Lmono-454-LGC-1	0.01	572.64	2	1	2	1
Lmono-454-LGC-2	0.01	319.50	2	1	2	1
Lmono-454-LGC-3	0.01	484.93	2	1	2	1
Lmono-454-NMIA-1	0.00	55.95	2	1	2	1
Lmono-LGC-Sanger-Clones	0.00	5.13	2	1	2	1
Lmono-NIST-Sanger-Clones	0.01	8.38	2	1	2	1
Consensus	0.01	1289.67	2	1	2	1

4 Appendix

Full List of False Positive Variants

All variants called by the pipelines used during the pipeline validation along with the suspected caus of the variant determined by manual investigation (see Biologically Conserved Positions Section above). The following abbreviations were used in Tables S4 and S5: Org - Organism, Plat - sequencing platform, Rep - replicate, Map - read mapping algorithm, Var - variant calling algorithm, POS - base position relative to the reference, DP - coverage, QUAL - confidence in variant call assigned by variant calling algorithm, MQ - mapping quality score assigned by mapping algorithm, FS - fisher strand bias test statistic, Cause - hypothesized cause of false positive variant call. See supplemental manuscript methods section for mapping algorithm and variant calling algorithm descriptions.

Table S4: ***E. coli*** **Pipeline Comparison** Characteristics of variant calls for different bioinformatic pipelines.

Org	Plat	Lab	Rep	Map	Var	POS	DP	QUAL	MQ	FS	Cause
Ecoli	454	LGC	1	BWA	GATK	324	250	443.77	60.00	47.88	End of read
Ecoli	454	LGC	1	TMAP	GATK	324	250	432.77	88.54	60.26	End of read
Ecoli	454	LGC	1	BWA	GATK	325	250	308.77	60.00	53.48	End of read
Ecoli	454	LGC	1	TMAP	GATK	325	250	309.77	88.54	50.67	End of read
Ecoli	454	LGC	1	BWA	SAMtools	396	2551	81.00	60.00		End of read
Ecoli	454	LGC	1	TMAP	SAMtools	396	3013	37.00	56.00		End of read
Ecoli	454	LGC	1	BWA	GATK	940	19	215.77	60.00	28.54	Non-target region
Ecoli	454	LGC	1	TMAP	GATK	940	21	179.77	80.15	28.54	Non-target region
Ecoli	454	LGC	1	BWA	GATK	959	250	1222.77	60.00	9.12	End of read
Ecoli	454	LGC	2	BWA	GATK	106	250	235.77	60.00	0.00	End of read
Ecoli	454	LGC	2	TMAP	GATK	106	250	34.77	68.90	0.00	End of read
Ecoli	454	LGC	2	BWA	GATK	959	250	795.77	59.98	28.04	End of read
Ecoli	454	LGC	3	BWA	GATK	324	250	231.77	59.83	40.63	End of read
Ecoli	454	LGC	3	TMAP	GATK	324	250	739.77	88.54	63.25	End of read
Ecoli	454	LGC	3	BWA	GATK	325	250	556.77	59.83	60.23	End of read
Ecoli	454	LGC	3	TMAP	GATK	325	250	498.77	88.54	36.85	End of read
Ecoli	454	LGC	3	BWA	GATK	348	250	741.77	59.92	11.62	End of read
Ecoli	454	LGC	3	BWA	SAMtools	417	1032	22.00	60.00		Homopolymer
Ecoli	454	LGC	3	TMAP	SAMtools	417	1020	32.00	58.00		Homopolymer
Ecoli	454	LGC	3	BWA	GATK	940	9	91.05	60.00	0.00	Non-target region
Ecoli	454	LGC	3	TMAP	GATK	940	14	194.29	82.41	0.00	Non-target region
Ecoli	454	NMIA	1	TMAP	GATK	313	250	5630.77	80.26	453.68	Strand bias
Ecoli	454	NMIA	1	TMAP	GATK	508	250	1160.77	83.71	0.00	End of read
Ecoli	454	NMIA	1	TMAP	GATK	509	250	1208.77	83.71	0.00	End of read
Ecoli	454	NMIA	1	TMAP	GATK	510	250	1275.77	83.71	0.00	End of read
Ecoli	454	NMIA	1	TMAP	GATK	514	250	1185.77	83.71	0.00	End of read
Ecoli	454	NMIA	1	TMAP	SAMtools	514	6337	5.46	59.00		End of read
Ecoli	454	NMIA	1	TMAP	GATK	901	208	8061.77	84.22	0.00	Non-target region
Ecoli	454	NMIA	1	TMAP	GATK	904	208	8023.77	84.22	0.00	Non-target region
Ecoli	454	NMIA	1	TMAP	GATK	934	250	8711.77	71.76	0.00	Non-target region
Ecoli	454	NMIA	1	TMAP	GATK	935	250	8708.77	71.76	0.00	Non-target region
Ecoli	454	NMIA	1	TMAP	GATK	938	250	8620.77	71.71	0.00	Non-target region
Ecoli	454	NMIA	1	TMAP	SAMtools	938	2747	9.54	56.00		Non-target region
Ecoli	454	NMIA	1	TMAP	GATK	939	250	8619.77	71.71	0.00	Non-target region
Ecoli	454	NMIA	1	TMAP	SAMtools	939	2747	15.20	60.00		Non-target region
Ecoli	454	NMIA	1	TMAP	SAMtools	941	2747	9.52	55.00		Non-target region
Ecoli	ION	NIMC	1	TMAP	SAMtools	1463	169	22.50	60.00		End of reference

Ecoli	Sanger	NIST	1	TMAP	SAMtools	1463	29	139.00	60.00		End of reference
Ecoli	Sanger	NIST	1	TMAP	SAMtools	1464	29	214.00	60.00		End of reference

Table S5: ***L. monocytogenes*** **Positions Pipeline Comparison**
Characteristics of variant calls for different bioinformatic pipelines.

Org	Plat	Lab	Rep	Map	Var	POS	DP	QUAL	MQ	FS	Cause
Lmono	454	LGC	1	BWA	GATK	315	250	3384.77	51.77	1.33	Contaminants
Lmono	454	LGC	1	BWA	GATK	328	250	3396.77	51.77	1.33	Contaminants
Lmono	454	LGC	1	BWA	GATK	346	250	149.77	59.51	37.09	End of read
Lmono	454	LGC	1	BWA	GATK	347	250	413.77	59.51	51.04	End of read
Lmono	454	LGC	1	TMAP	GATK	347	250	130.77	91.19	40.53	End of read
Lmono	454	LGC	1	BWA	GATK	555	144	69.77	57.88	2.20	Contaminants
Lmono	454	LGC	1	BWA	GATK	587	144	83.77	57.88	2.17	Contaminants
Lmono	454	LGC	1	BWA	GATK	677	145	122.77	59.80	2.17	Contaminants
Lmono	454	LGC	1	BWA	GATK	700	145	119.77	59.80	2.17	Contaminants
Lmono	454	LGC	1	BWA	GATK	703	145	119.77	59.80	2.17	Contaminants
Lmono	454	LGC	1	BWA	GATK	712	145	105.77	59.80	2.17	Contaminants
Lmono	454	LGC	1	BWA	GATK	716	145	94.77	59.80	2.17	Contaminants
Lmono	454	LGC	1	BWA	GATK	729	145	118.77	59.80	2.17	Contaminants
Lmono	454	LGC	1	BWA	GATK	738	145	122.77	59.80	2.17	Contaminants
Lmono	454	LGC	1	BWA	GATK	740	145	122.77	59.80	2.17	Contaminants
Lmono	454	LGC	1	BWA	GATK	741	145	161.77	59.80	2.12	Contaminants
Lmono	454	LGC	1	BWA	GATK	742	145	119.77	59.80	2.17	Contaminants
Lmono	454	LGC	1	BWA	GATK	743	145	119.77	59.80	2.17	Contaminants
Lmono	454	LGC	1	BWA	GATK	753	145	114.77	59.80	2.17	Contaminants
Lmono	454	LGC	1	BWA	GATK	757	145	120.77	59.80	2.17	Contaminants
Lmono	454	LGC	1	BWA	GATK	963	122	701.29	60.00	0.00	Contaminants
Lmono	454	LGC	1	TMAP	GATK	963	21	82.31	74.82	0.00	Contaminants
Lmono	454	LGC	1	BWA	GATK	1047	250	3748.77	59.89	4.09	Contaminants
Lmono	454	LGC	1	BWA	GATK	1055	250	3571.77	59.89	7.83	Contaminants
Lmono	454	LGC	1	BWA	GATK	1072	250	2637.77	59.92	18.48	Contaminants
Lmono	454	LGC	1	BWA	GATK	1077	249	2161.77	59.92	18.66	Contaminants
Lmono	454	LGC	1	BWA	GATK	1192	250	4830.77	59.65	8.22	Contaminants
Lmono	454	LGC	1	BWA	GATK	1201	250	4741.77	59.65	5.67	Contaminants
Lmono	454	LGC	1	BWA	GATK	1208	250	4714.77	59.65	8.23	Contaminants
Lmono	454	LGC	1	BWA	GATK	1213	250	4701.77	59.65	8.18	Contaminants
Lmono	454	LGC	1	BWA	GATK	1304	250	4652.77	60.00	6.46	Contaminants
Lmono	454	LGC	1	BWA	GATK	1307	250	4789.77	60.00	8.27	Contaminants
Lmono	454	LGC	1	BWA	GATK	1318	250	4710.77	60.00	8.42	Contaminants
Lmono	454	LGC	1	BWA	GATK	1321	250	4641.77	60.00	8.42	Contaminants
Lmono	454	LGC	1	BWA	GATK	1329	250	4555.77	60.00	10.65	Contaminants
Lmono	454	LGC	1	BWA	GATK	1356	250	4453.77	60.00	8.63	Contaminants
Lmono	454	LGC	2	BWA	GATK	346	250	102.77	60.00	32.12	End of read
Lmono	454	LGC	2	TMAP	GATK	346	250	294.77	91.54	40.01	End of read
Lmono	454	LGC	2	BWA	GATK	347	250	255.77	60.00	42.21	End of read
Lmono	454	LGC	2	BWA	GATK	370	250	67.77	60.00	8.83	End of read
Lmono	454	LGC	2	BWA	GATK	963	111	302.48	60.00	0.00	Non-target region
Lmono	454	LGC	2	TMAP	GATK	963	10	78.77	67.25	0.00	Non-target region
Lmono	454	LGC	3	BWA	GATK	346	250	84.77	60.00	31.53	End of read
Lmono	454	LGC	3	TMAP	GATK	346	250	60.77	91.33	32.84	End of read
Lmono	454	LGC	3	BWA	GATK	347	250	238.77	60.00	39.33	End of read

Lmono	454	LGC	3	TMAP	GATK	347	250	533.77	91.33	56.41	End of read
Lmono	454	LGC	3	BWA	GATK	963	189	811.29	60.00	0.00	Non-target region
Lmono	454	LGC	3	BWA	SAMtools	963	189	9.51	60.00		Non-target region
Lmono	454	LGC	3	TMAP	GATK	963	22	48.78	65.69	0.00	Non-target region
Lmono	454	NMIA	1	TMAP	GATK	330	250	5990.77	79.12	466.25	Strand bias
Lmono	454	NMIA	1	TMAP	GATK	334	250	5973.77	79.12	514.24	Strand bias
Lmono	454	NMIA	1	TMAP	GATK	335	250	5199.77	79.12	512.93	Strand bias
Lmono	454	NMIA	1	BWA	GATK	381	250	37.77	60.00	13.82	End of read
Lmono	454	NMIA	1	TMAP	GATK	533	249	1662.77	67.08	0.00	End of read
Lmono	454	NMIA	1	TMAP	GATK	932	94	3555.77	75.44	0.00	Non-target region
Lmono	454	NMIA	1	TMAP	GATK	936	92	3557.77	75.89	0.00	Non-target region
Lmono	454	NMIA	1	TMAP	GATK	954	250	8461.77	77.83	0.00	Non-target region
Lmono	454	NMIA	1	TMAP	GATK	957	250	8402.77	77.83	0.00	Non-target region
Lmono	454	NMIA	1	TMAP	GATK	961	250	8422.77	77.83	0.00	Non-target region
Lmono	454	NMIA	1	TMAP	GATK	962	250	8332.77	77.83	0.00	Non-target region
Lmono	454	NMIA	1	TMAP	GATK	963	250	8246.77	77.83	0.00	Non-target region
Lmono	ION	NIST	1	BWA	GATK	792	259	132.77	60.00	54.38	Strand bias
Lmono	ION	NIST	1	TMAP	GATK	792	275	323.77	85.79	69.53	Strand bias
Lmono	Sanger	LGC	1	BWA	SAMtools	390	81	25.50	60.00		End of read
Lmono	Sanger	LGC	1	BWA	SAMtools	1409	44	13.70	60.00		End of read
Lmono	Sanger	LGC	1	TMAP	SAMtools	1505	41	71.20	60.00		End of reference
Lmono	Sanger	LGC	1	TMAP	SAMtools	1506	41	71.20	60.00		End of reference
Lmono	Sanger	NIST	1	BWA	SAMtools	865	74	76.50	60.00		End of read
Lmono	Sanger	NIST	1	TMAP	SAMtools	865	68	77.50	59.00		End of read
Lmono	Sanger	NIST	1	BWA	GATK	867	67	264.77	60.00	0.00	End of read
Lmono	Sanger	NIST	1	BWA	SAMtools	867	67	10.40	60.00		End of read
Lmono	Sanger	NIST	1	TMAP	GATK	867	64	249.77	96.41	0.00	End of read
Lmono	Sanger	NIST	1	TMAP	SAMtools	867	64	12.30	59.00		End of read
Lmono	Sanger	NIST	1	TMAP	SAMtools	1504	35	214.00	60.00		End of read

Contaminants - BLAST results

BLAST reports for representative sequences of reads responsible for false positive variant calls in the LGC *L. monocytogenes* “454” rep 1 dataset.

```
BLASTN 2.2.29+
Reference: Zheng Zhang, Scott Schwartz, Lukas Wagner, and
Webb Miller (2000), "A greedy algorithm for aligning DNA
sequences", J Comput Biol 2000; 7(1-2):203-14.


RID: KH9SY3U8014


Database: Representative Chromosomes
           2,857 sequences; 5,609,140,793 total letters
Query=
Length=558


                                                                  Score     E
Sequences producing significant alignments:                      (Bits)  Value
```

```
ref|NC_000913.3|  Escherichia coli str. K-12 substr. MG1655, c...    979   0.0
ref|NC_018658.1|  Escherichia coli O104:H4 str. 2011C-3493 chr...    979   0.0
ref|NC_017634.1|  Escherichia coli O83:H1 str. NRG 857C chromo...    979   0.0
ref|NC_011751.1|  Escherichia coli UMN026 chromosome, complete...    979   0.0
ref|NC_011750.1|  Escherichia coli IAI39 chromosome, complete ...    979   0.0
ref|NC_011740.1|  Escherichia fergusonii ATCC 35469 chromosome...    979   0.0
ref|NC_007384.1|  Shigella sonnei Ss046 chromosome, complete g...    979   0.0
ref|NC_002695.1|  Escherichia coli O157:H7 str. Sakai chromoso...    979   0.0
ref|NC_004337.2|  Shigella flexneri 2a str. 301 chromosome, co...    974   0.0
ref|NC_007613.1|  Shigella boydii Sb227 chromosome, complete g...    974   0.0

ALIGNMENTS
>ref|NC_000913.3| Escherichia coli str. K-12 substr. MG1655, complete genome
Length=4641652

 Features in this part of subject sequence:
   rRNA-16S ribosomal RNA of rrnH operon

 Score =   979 bits (530),  Expect = 0.0
 Identities = 539/543 (99%), Gaps = 2/543 (0%)
 Strand=Plus/Plus

Query  3       CCTGATGCAGCCATGCCGCGTGTATGAAGAAGGCTTACGGGTTGT-AAGTACGTTTCAGC  61
               |||||||||||||||||||||||||||||||||| | |||||||| |||||| |||||||
Sbjct  224155  CCTGATGCAGCCATGCCGCGTGTATGAAGAAGGCCTTCGGGTTGTAAAGTAC-TTTCAGC  224213

Query  62      GGGGAGGAAGGGAGTAAAGTTAATACCTTTGCTCATTGACGTTACCCGCAGAAGAAGCAC  121
               ||||||||||||||||||||||||||||||||||||||||||||||||||||||||||||
Sbjct  224214  GGGGAGGAAGGGAGTAAAGTTAATACCTTTGCTCATTGACGTTACCCGCAGAAGAAGCAC  224273

Query  122     CGGCTAACTCCGTGCCAGCAGCCGCGGTAATACGGAGGGTGCAAGCGTTAATCGGAATTA  181
               ||||||||||||||||||||||||||||||||||||||||||||||||||||||||||||
Sbjct  224274  CGGCTAACTCCGTGCCAGCAGCCGCGGTAATACGGAGGGTGCAAGCGTTAATCGGAATTA  224333

Query  182     CTGGGCGTAAAGCGCACGCAGGCGGTTTGTTAAGTCAGATGTGAAATCCCCGGGCTCAAC  241
               ||||||||||||||||||||||||||||||||||||||||||||||||||||||||||||
Sbjct  224334  CTGGGCGTAAAGCGCACGCAGGCGGTTTGTTAAGTCAGATGTGAAATCCCCGGGCTCAAC  224393

Query  242     CTGGGAACTGCATCTGATACTGGCAAGCTTGAGTCTCGTAGAGGGGGGTAGAATTCCAGG  301
               ||||||||||||||||||||||||||||||||||||||||||||||||||||||||||||
Sbjct  224394  CTGGGAACTGCATCTGATACTGGCAAGCTTGAGTCTCGTAGAGGGGGGTAGAATTCCAGG  224453

Query  302     TGTAGCGGTGAAATGCGTAGAGATCTGGAGGAATACCGGTGGCGAAGGCGGCCCCCTGGA  361
               ||||||||||||||||||||||||||||||||||||||||||||||||||||||||||||
Sbjct  224454  TGTAGCGGTGAAATGCGTAGAGATCTGGAGGAATACCGGTGGCGAAGGCGGCCCCCTGGA  224513

Query  362     CGAAGACTGACGCTCAGGTGCGAAAGCGTGGGGAGCAAACAGGATTAGATACCCTGGTAG  421
               ||||||||||||||||||||||||||||||||||||||||||||||||||||||||||||
Sbjct  224514  CGAAGACTGACGCTCAGGTGCGAAAGCGTGGGGAGCAAACAGGATTAGATACCCTGGTAG  224573

Query  422     TCCACGCCGTAAACGATGTCGACTTGGAGGTTGTGCCCTTGAGGCGTGGCTTCCGGAGCT  481
               ||||||||||||||||||||||||||||||||||||||||||||||||||||||||||||
Sbjct  224574  TCCACGCCGTAAACGATGTCGACTTGGAGGTTGTGCCCTTGAGGCGTGGCTTCCGGAGCT  224633
```

```
Query  482     AACGCGTTAAGTCGACCGCCTGGGGAGTACGGCCGCAAGGTTAAAACTCAAATGAATTGA  541
               ||||||||||||||||||||||||||||||||||||||||||||||||||||||||||||
Sbjct  224634  AACGCGTTAAGTCGACCGCCTGGGGAGTACGGCCGCAAGGTTAAAACTCAAATGAATTGA  224693

Query  542     CGG  544
               |||
Sbjct  224694  CGG  224696


  Database: Representative Chromosomes
    Posted date:  Mar 21, 2014  12:17 AM
  Number of letters in database: 5,609,140,793
  Number of sequences in database:  2,857

Lambda      K        H
    1.33    0.621     1.12
Gapped
Lambda      K        H
    1.28    0.460    0.850
Matrix: blastn matrix:1 -2
Gap Penalties: Existence: 0, Extension: 0
Number of Sequences: 2857
Number of Hits to DB: 6177
Number of extensions: 6
Number of successful extensions: 6
Number of sequences better than 10: 1
Number of HSP's better than 10 without gapping: 0
Number of HSP's gapped: 3
Number of HSP's successfully gapped: 3
Length of query: 558
Length of database: 5609140793
Length adjustment: 30
Effective length of query: 528
Effective length of database: 5609055083
Effective search space: 2961581083824
Effective search space used: 2961581083824
A: 0
X1: 13 (25.0 bits)
X2: 32 (59.1 bits)
X3: 54 (99.7 bits)
S1: 13 (25.1 bits)
S2: 21 (39.9 bits)
```
